# Droplet slipperiness despite surface heterogeneity at molecular scale

**DOI:** 10.1038/s41557-023-01346-3

**Published:** 2023-10-23

**Authors:** Sakari Lepikko, Ygor Morais Jaques, Muhammad Junaid, Matilda Backholm, Jouko Lahtinen, Jaakko Julin, Ville Jokinen, Timo Sajavaara, Maria Sammalkorpi, Adam S. Foster, Robin H. A. Ras

**Affiliations:** 1 Department of Applied Physics, Aalto University, Espoo, Finland; 2 Centre of Excellence in Life-Inspired Hybrid Materials (LIBER), Aalto University, Espoo, Finland; 3 Department of Chemistry and Materials Science, Aalto University, Espoo, Finland; 4 Department of Physics, University of Jyväskylä, Jyväskylä, Finland; 5 Department of Bioproducts and Biosystems, Aalto University, Espoo, Finland; 6 Nano Life Science Institute (WPI-NanoLSI), Kanazawa University, Kakuma-machi, Kanazawa, Japan

**Keywords:** Surface chemistry, Wetting

## Abstract

Friction determines whether liquid droplets slide off a solid surface or stick to it. Surface heterogeneity is generally acknowledged as the major cause of increased contact angle hysteresis and contact line friction of droplets. Here we challenge this long-standing premise for chemical heterogeneity at the molecular length scale. By tuning the coverage of self-assembled monolayers (SAMs), water contact angles change gradually from about 10° to 110° yet contact angle hysteresis and contact line friction are low for the low-coverage hydrophilic SAMs as well as high-coverage hydrophobic SAMs. Their slipperiness is not expected based on the substantial chemical heterogeneity of the SAMs featuring uncoated areas of the substrate well beyond the size of a water molecule as probed by metal reactants. According to molecular dynamics simulations, the low friction of both low- and high-coverage SAMs originates from the mobility of interfacial water molecules. These findings reveal a yet unknown and counterintuitive mechanism for slipperiness, opening new avenues for enhancing the mobility of droplets.

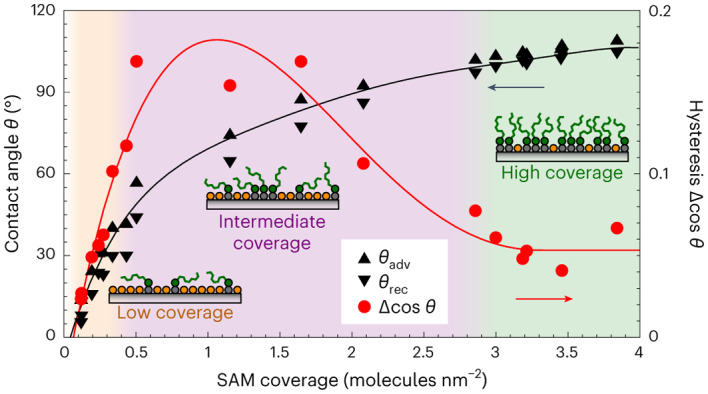

## Abstract (web summary)

Surface heterogeneity is generally acknowledged as the major cause of liquid–solid friction, affecting whether droplets slide off the surface or stick to it. Now, a model surface of self-assembled monolayers has been used to investigate how molecular-scale surface heterogeneity affects water contact angle hysteresis and contact line friction. The high-coverage hydrophobic surface is slippery, as—counter-intuitively—is the low-coverage hydrophilic surface.

## Main

Friction between a solid surface and water is present in everyday life in many ways. Surfaces such as regular window glass and rose petals have high friction resulting in droplets sticking even to vertical surfaces. Other surfaces, like lotus leaves and Teflon-coated kitchenware, have low friction allowing droplets to slide off even at small tilt angles. This latter feature of slipperiness is highly sought after^1–3^, for applications such as self-cleaning^4^, anti-icing^5^, microfluidics^6^ and heat transfer^7^.

Four distinct forms of droplet friction have been identified: contact line friction (CLF)^8–11^, viscous dissipation^12^, air resistance^13^, and electrostatic forces^14^. In general, CLF *F*_μ_ is the cause of the static friction of the droplet^15^ while the other forces arise only when the droplet has already started moving. CLF is shown to relate to droplet contact angles (CAs) via the following equation:

1 $${F}_{{\upmu }}={AR}\gamma \left(\cos {\theta }_{\text{REC}}-\cos {\theta }_{\text{ADV}}\right)$$

where *A* is a droplet contact area shape parameter, *R* is droplet size parameter, *γ* is liquid surface tension, and *θ*_REC_ and *θ*_ADV_ are receding contact angle (RCA) and advancing contact angle (ACA), respectively^9,10,16^. The difference between *θ*_REC_ and *θ*_ADV_ is called contact angle hysteresis (CAH)^17^, and its magnitude is directly related to the CLF.

It is widely acknowledged that droplet CAH originates from heterogeneities in surface topography and chemistry, as suggested theoretically in the early work by Pease^18^, Johnson and Dettre^19^, and Joanny and de Gennes^8^, and demonstrated experimentally for chemical patterns and topographies at the micrometre scale by Furuta et al.^20^ and Reyssat and Quéré^21^. The common understanding is that this effect of surface heterogeneities is valid in general, thus also when length scales are reduced to molecular scale, and Fadeev et al.^22,23^ and Bittoun et al.^24^ have experimentally shown that molecular-level heterogeneity of the surface affects CAH.

**Fig. 1:**
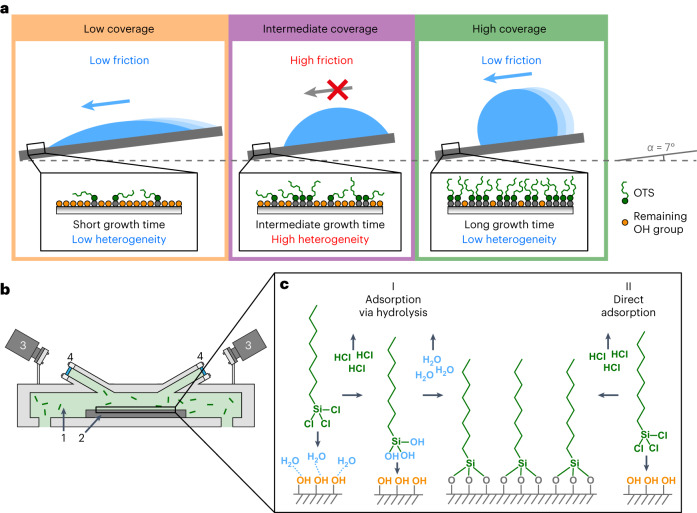
Controlling CLF by tuning surface hydrophobicity with OTS SAM. **a**, We observe that CLF depends on heterogeneity of the SAM and has lowest values both at low and high SAM coverage regimes, and highest values at intermediate coverages. **b**, Schematics of the vapour deposition reactor chamber for growing SAMs, featuring operando spectroscopic ellipsometry for continuous in situ monitoring the growth of SAM films. Numbers represent (1) the reactor chamber with OTS molecules (illustrated by short green lines), (2) the substrate, (3) operando ellipsometer and (4) viewports of the reactor chamber. **c**, Adsorption mechanisms of OTS SAM on hydroxyl-rich surfaces: (I) OTS molecules first hydrolyse using surface-bound water. The hydrolysed OTS molecules then bond covalently to surface hydroxyl groups. (II) Alternatively, OTS molecules may form covalent bonds directly to surface hydroxyl groups.

In this Article, we quantify systematically how molecular-scale chemical heterogeneity affects CAH and droplet CLF. As a model system, we use octyltrichlorosilane (OTS) self-assembled monolayer (SAM) grown on a flat, OH-terminated SiO_2_ surface. By varying the SAM growth time, we gradually varied its coverage (that is, its areal density) from OH-group-rich hydrophilic SiO_2_ substrate to a hydrophobic high-coverage SAM. CLF was observed to be high for the intermediate-coverage SAM that has a large fraction of OH groups and is thus chemically the most heterogeneous surface, and low for both low- and high-coverage SAMs that are chemically less heterogeneous yet still not completely homogeneous either (Fig. 1a). The finding that hydrophilic low-coverage SAM has low CLF is counterintuitive, and we performed molecular dynamics (MD) simulations for better understanding the SAM structure and droplet friction mechanisms. These findings help improve the performance of repellent coatings that benefit from low CLF. As an example, we achieved record-low CLF by minimizing chemical heterogeneity of a nanostructured superhydrophobic surface.

## Results and discussion

We selected to grow SAMs from the vapour phase using a uniquely designed atomic layer deposition(ALD) reactor to allow excellent control of reaction conditions (precise OTS dosing, water content, temperature and so on) and continuous in situ observation of SAM growth with operando spectroscopic ellipsometry (Fig. 1b), enabling unprecedented precise tuning of SAM coverage. Chlorosilane SAM growth is known to be critically dependent on the water content in the reaction environment where a too high water content results in undesired chlorosilane polymerization and aggregate formation while dry conditions with only trace amounts of water produces smoother and more uniform SAMs^25,26^. Therefore, optimal monolayer growth is achieved in controlled dry conditions either via hydrolysis due to surface-bound water^25^ or direct adsorption to surface hydroxyl groups^26^ (Fig. 1c and Supplementary Note 1).

### Growth and structure of SAM surfaces

**Fig. 2:**
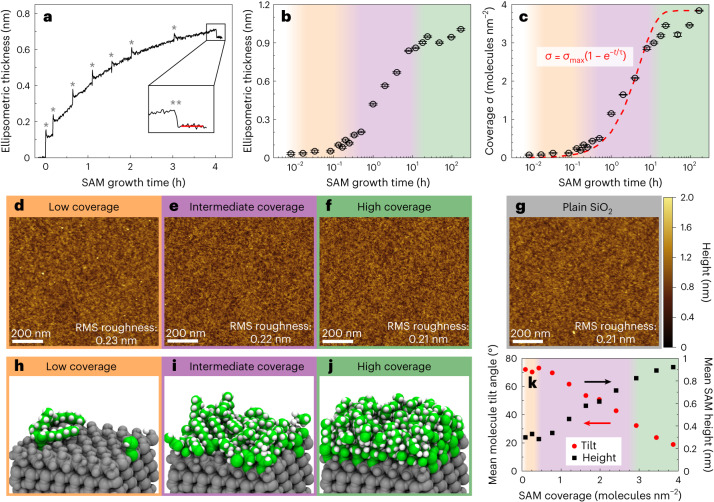
From sparsely to densely coated OTS SAMs. **a**, OTS SAM thickness monitored by ellipsometry for a 4 h run. Steps of reactor evacuation and introduction of new OTS vapour dose are marked with asterisk. Final SAM thickness is obtained by averaging thickness over the region marked with red line after removal of physisorbed OTS molecules with the reactor final purge (**; details in Supplementary Note 2). **b**, Final ellipsometric thickness of SAM surfaces as function of growth time. **c**, SAM coverage calculated from ellipsometer data. The red dashed line represents standard monolayer growth model fit to the data. In **b** and **c**, each data point represents average thickness/coverage value after the final purge of a single prepared sample surface, and error bars represent the standard deviation and do not include possible systematic error due to OTS SAM refractive index/thickness assumption (Supplementary Note 3). Orange/purple/green background shade represents low/intermediate/high coverage regimes, respectively. **d**–**g**, AFM topography images of low-coverage (0.1 molecules nm^−^^2^) (**d**), intermediate-coverage (0.5 molecules nm^−^^2^) (**e**) and high-coverage (3.2 molecules nm^−^^2^) (**f**) SAM in comparison with reference SiO_2_ without SAM (**g**). The root mean square (RMS) roughness of the surfaces is shown in the bottom right corners of the images. Height scale is common in **d**–**g**. **h**–**j**, Snapshots of simulated OTS molecules adsorbed on the SiO_2_ substrate with representative low-coverage (0.2 molecules nm^−^^2^) (**h**), intermediate-coverage (2.0 molecules nm^−^^2^) (**i**) and high-coverage (3.4 molecules nm^−^^2^) (**j**) SAMs. **k**, Mean molecule tilt angle from surface normal and SAM height in the MD simulations as a function of the SAM coverage. Average is calculated for all molecules over the simulation time period (Supplementary Note 7). Source data

We prepared 20 SAM surfaces with growth times from 30 s to 168 h and monitored their growth with operando ellipsometry (Supplementary Note 2). Figure 2a shows how SAM thickness evolves during a 4 h growth (Supplementary Fig. 1 shows other growth times; Supplementary Note 3 gives details of ellipsometer data and error analysis). The growth is initially fast and slows down as SAM coverage increases. The final SAM thickness depends on the total growth time, and tens of hours are needed for OTS SAM to reach maximal growth (Fig. 2b). The obtained ellipsometry data can be used to calculate SAM coverage in terms of adsorbed molecules per unit area (Fig. 2c and Supplementary Note 3). The growth does not reach a clear saturation even after 168 h and resembles standard monolayer growth model (Supplementary Note 4), although the real growth is slightly less steep than predicted by the model. We checked that surface OH groups not yet reacted with SAM and the amount of surface-bound water remain stable in the deposition reactor (Supplementary Note 5), so the slowing down of the growth could be due to structural changes required for denser packing of octyl chains. However, Fourier transform infrared spectroscopy (FTIR) shows that the denser packing does not substantially increase alkyl chain crystallinity, and the SAM has an amorphous structure still at 3.9 molecules nm^−^^2^ coverage (Supplementary Note 6). All SAM surfaces also maintain the smoothness of the substrate surface based on atomic force microscopy (AFM; Fig. 2d–g), implying spatially uniform growth above nanometre scale without evidence of aggregate formation, meaning that surface heterogeneity remains at the molecular scale only.

To further investigate the molecular-level structural properties of OTS SAM surfaces, we performed MD simulations of OTS SAMs with varying coverage. The simulations reveal three distinct growth regimes (Fig. 2h–k and Supplementary Note 7). In the low-coverage regime below 0.5 molecules nm^−^^2^, the alkyl chains of the OTS molecules prefer to lie flat on the surface and have a high average tilt angle close to 80°. Above 0.5 molecules nm^−^^2^, each alkyl chain has less free space around it, and some of the chains lay on top of other chains, decreasing average tilt angle to 50° (Supplementary Fig. 6). At coverages exceeding 2.9 molecules nm^−^^2^, alkyl chains have little room, and most of them are forced to near-vertical orientation with average tilt angle below 10°. This gradual decrease of tilt angle increases height of the SAM (Fig. 2k and Supplementary Note 7), which corresponds well with the observed thickness of real SAM surfaces.

### Quantification of OH vacancies of SAM surfaces

**Fig. 3:**
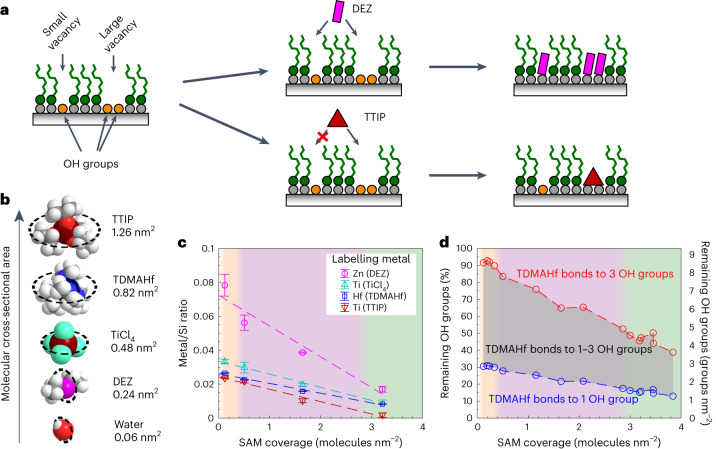
Labelling of SAM OH vacancies by metal compounds for probing the chemical heterogeneity at molecular length scales. **a**, A schematic representation of how chemical heterogeneity at molecular length scales is probed using the reaction of metal compounds to the surface OH groups. The reaction will be sterically hindered if the incoming metal reactant is larger than the accessible space to reach the oxide surface confined by OTS molecules. Metal reactants of different sizes allow for probing the size of accessible space. Alkyl chain tilt is ignored for clarity. **b**, Structures and sizes of metal compounds used to mark defect sites in this study. The molecular cross-sectional area is estimated on the basis of the area of smallest circle through which the molecule can pass. The size of water molecule is shown for reference. **c**, Metal-to-Si ratios of the four metal compounds deposited on the SAM surfaces. The dashed lines represent linear fits to data points. Data point error bars represent the standard deviation of the mean obtained from three different measurement locations. **d**, Estimated range of remaining OH groups as function of SAM coverage based on RBS measurement of TDMAHf-labelled SAMs. Red and blue data points represent the limits where each TDMAHf has bonded to three OH groups and one OH group, respectively, and the darkened area represents the possible values between these two limits. In **c** and **d** the orange/purple/green background shade represents low-/intermediate-/high-coverage regimes, respectively. Source data

To probe surface chemical heterogeneity at the molecular scale, we aim to quantify the fraction of the substrate that remains uncoated, that is, OH vacancies of the SAM surface that impart hydrophilic character to the SAM. Here we quantify the remaining, accessible surface OH vacancies in SAMs by reaction with metal compounds. Alkyls from the SAM act as a resist against these metal compounds, which can adsorb only to the accessible OH groups of the surface^27,28^ (Fig. 3a and Supplementary Note 8). As such, metal reactants are used here for ‘labelling’ these OH groups, and the labels can be quantified with surface-sensitive elemental analysis techniques. This methodology allows both quantification of the label density, that is, number of OH vacancies per area, and also determination of the size of the OH vacancy at molecular scale. In case the size of the vacancy is small, it will react only with a small labelling molecule and not with a larger one. We selected four metal compounds with increasing size: diethyl zinc (DEZ), titanium tetrachloride (TiCl_4_) tetrakis(dimethylamido) hafnium(IV) (TDMAHf) and titanium tetraisopropoxide (TTIP) (Fig. 3b).

The adsorption of labelling molecules was detected using X-ray photoelectron spectroscopy (XPS), and Fig. 3c shows the results (details in Supplementary Note 9). The concentration of all labelling molecules decreases rather linearly as SAM coverage increases, showing that there are fewer OH vacancies remaining on the surface. DEZ has the highest concentration as it is the smallest of the metal reactants and can react with up to two adjacent OH groups (Supplementary Fig. 7), followed by other labelling molecules according to their sizes and capability to react with up to three adjacent OH groups (Supplementary Fig. 7). For the three smaller labelling molecules, the relative decrease of metal-to-Si ratio is comparable and drops 70–80% when SAM coverage increases from 0.1 to 3.2 molecules nm^−^^2^. Consequently, there is still a large fraction of OH vacancy sites remaining even for SAM coverage of 3.2 molecules nm^−^^2^. Ti-to-Si ratio for TTIP drops over 90%, implying that larger vacancies disappear faster than smaller ones, as can be expected for spatially uniformly grown SAMs.

The TDMAHf-labelled samples were further analysed with Rutherford backscattering (RBS) to obtain the areal density of the labelling molecules (Supplementary Note 10 and Supplementary Fig. 10). On a reference SiO_2_ surface without a SAM there are 3.1 TDMAHf labels nm^−^^2^, which corresponds up to 9.3 OH groups nm^−^^2^ when each TDMAHf has bonded to three OH groups, meaning that nearly all surface Si atoms have an OH group. By coating the substrate with SAM, the share of accessible OH groups decreases (Fig. 3d). As the SAM density is increased, fewer TDMAHf labels can find room to bond to three adjacent surface OH groups, and the average number of bonds between TDMAHf and OH groups decreases towards one. Determining the average number of bonds to OH groups for each SAM surface is beyond the scope of this article, and instead Fig. 3d shows the possible range of accessible OH groups for all studied SAM surfaces. We can conclude that the increase of SAM coverage reduces the amount of accessible OH groups. Within the probed coverage range, the lowest-coverage SAM (0.1 molecules nm^−^^2^) has reacted with at least 7% of the OH groups, and the highest coverage SAM (3.84 molecules nm^−^^2^) has still at least 13% of the OH groups remaining accessible for TDMAHf. All of the SAM surfaces from low to high coverage are thus heterogeneous containing alkyls as well as accessible OH groups.

### Wetting properties of chemically heterogeneous SAM

**Fig. 4:**
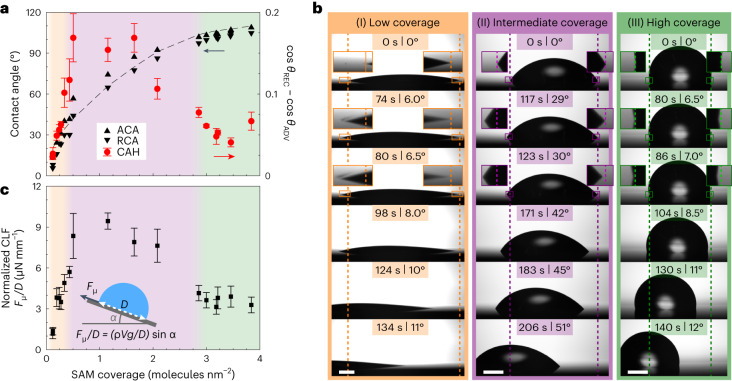
SAM wetting properties. **a**, ACA, RCA and CAH as function of OTS SAM coverage. The dashed line shows the CA prediction from Cassie’s law. The data points represent average values recorded from three locations, and error bars represent the standard deviation. Error bars for ACA and RCA are small and not shown. **b**, Snapshots of 10 μl droplets sliding on (I) low-coverage (0.1 molecules nm^−^^2^), (II) intermediate-coverage (0.5 molecules nm^−^^2^) and (III) high-coverage (3.2 molecules nm^−^^2^) SAMs. Time and surface tilt angle are shown in the top banner of each frame, and dashed lines show initial location of the droplet. Insets show magnifications of droplet front and rear edges. Second row of frames represents the last moment before sliding started. Scale bars, 1 mm. **c**, Normalized CLF (*F*_μ_/*D*) calculated from SAs. The data points represent average CLF values measured with droplet volumes of 5 μl, 10 μl, and 20 μl, each measured at three different locations. Error bars represent standard deviation of the average CLF. The inset shows how normalized CLF is obtained from SAs. In the equation *ρ* and *V* are droplet density and volume, respectively, *g* is gravitational constant and *α* is droplet SA. In **a** and **c** the orange/purple/green background shade represents low-/intermediate-/high-coverage regimes, respectively. Source data

The wetting properties of SAM surfaces were evaluated via water CA and sliding angle (SA) measurements. Plain SiO_2_ and low-coverage SAMs below 0.1 molecules nm^−^^2^ have high areal density of OH vacancies, and water spreads to form a film on these surfaces. Only above 0.1 molecules nm^−^^2^ coverage, water maintains a droplet shape, enabling measurements of ACA and RCA. Figure 4a shows how ACA and RCA gradually increase from 13° and 6° to 109° and 105°, respectively, as SAM coverage increases from 0.1 molecules nm^−^^2^ to 3.9 molecules nm^−^^2^. The increase is in line with MD simulations of CAs of droplets on OTS SAM surfaces (Supplementary Note 11 and Supplementary Fig. 12 and 13) and with Cassie’s law prediction if considering both SAM coverage and alkyl chain tilt (Supplementary Note 12). According to the observed CAs and Cassie’s law prediction, the surface is half covered by alkyl chains near a SAM coverage of 1 molecule nm^−^^2^. This is also the coverage regime where the highest CAH ($\cos {\theta }_{{\rm{REC}}}-\cos {\theta }_{{\rm{ADV}}}$) above 0.15 is observed. Below that regime, the surface is less heterogeneous as it is mostly uncoated SiO_2_, and above it is less heterogeneous as it is mostly covered by the SAM, resulting in a reduced CAH even below 0.05. Interestingly, CAH is approximately constant above a SAM coverage of 3 molecules nm^−^^2^ even though CAs still slightly increase as SAM coverage increases. We will return to this finding later.

**Extended Data Fig. 1:**
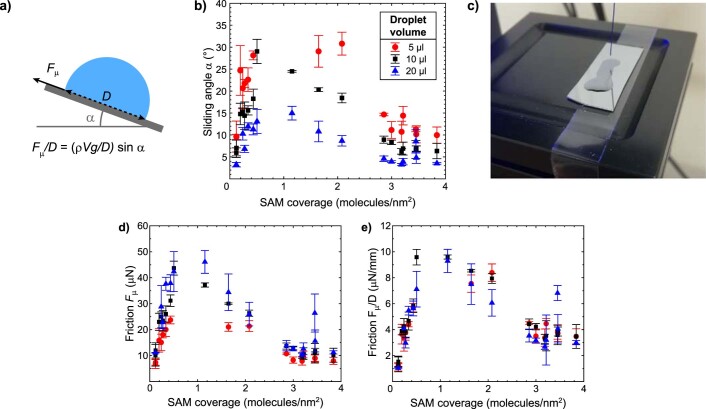
Sliding of water droplets on OTS SAM surfaces. **a)** Schematics of the sliding angle experiments. *F*_μ_ represents contact line friction, *D* droplet contact region diameter, *ρ* water density (*ρ* = 1.0 g cm^−3^), *V* droplet volume, *g* acceleration of gravity (*g* = 9.81 m/s^2^), and *α* tilt of the surface when the droplet starts sliding. **b)** Water droplet sliding angle as function of SAM coverage ranging from 0.1 molecules/nm^2^ to 3.8 molecules/nm^2^. Sliding angles were recorded with three different volumes: 5 µl, 10 µl and 20 µl. **c)** Droplet leaving a trailing film on a SAM with coverage below 0.1 molecules/nm^2^, meaning that sliding angle could not be determined below 0.1 molecules/nm^2^ coverage. **d)** Calculated contact line friction from the sliding angles as function of SAM coverage. **e)** Contact line friction normalized with droplet contact region diameter (*F*_μ_/*D*) as function of SAM coverage. In b), d) and e), each data point represents average value of three measurements made at different locations of the SAM surfaces, and error bars represent standard deviation. Source data

Droplet CLF was measured via SA measurements (Extended Data Fig. 1 and Supplementary Note 13). The intermediate-coverage SAMs have higher SAs than low- and high-coverage SAMs (Fig. 4b), similarly as CAH is highest in the intermediate-coverage regime. Figure 4c shows CLF obtained from SAs and normalized by droplet contact region diameter as shown in the figure inset. The low-coverage SAM with 0.1 molecules nm^−^^2^ has very low normalized CLF of only 1.2 μN mm^−1^ (corresponding to 6° SA for 10-μl droplet, Supplementary Video 2). This value is unintuitively low given that the surface is very hydrophilic and thus has high adhesion for water. It is comparable to other state-of-the-art slippery surfaces, such as polyethylene glycol-based hydrophilic surface^29^, hydrophobic slippery liquid-infused porous surfaces (SLIPS)^30,31^ and slippery omniphobic covalently attached liquid surfaces (SOCAL)^32–34^, and even to certain superhydrophobic surfaces^35,36^, although the large difference in contact area between low- and high-CA droplets results in different SAs. Increasing SAM coverage to the intermediate regime increases normalized CLF, reaching 8.4 μN mm^−1^ at 0.5 molecules nm^−^^2^ (corresponding to 28° SA for 10-μl droplet, Supplementary Video 3). Lower normalized CLF of 3.8 μN mm^−1^ is obtained again at high SAM coverage regime at 3.2 molecules nm^−^^2^ (corresponding to 6° SA for 10-μl droplet, Supplementary Video 4), and like with CAH, normalized CLF is approximately constant in coverage regime of 3–4 molecules nm^−^^2^.

### Molecular-level origin of SAM CLF

**Fig. 5:**
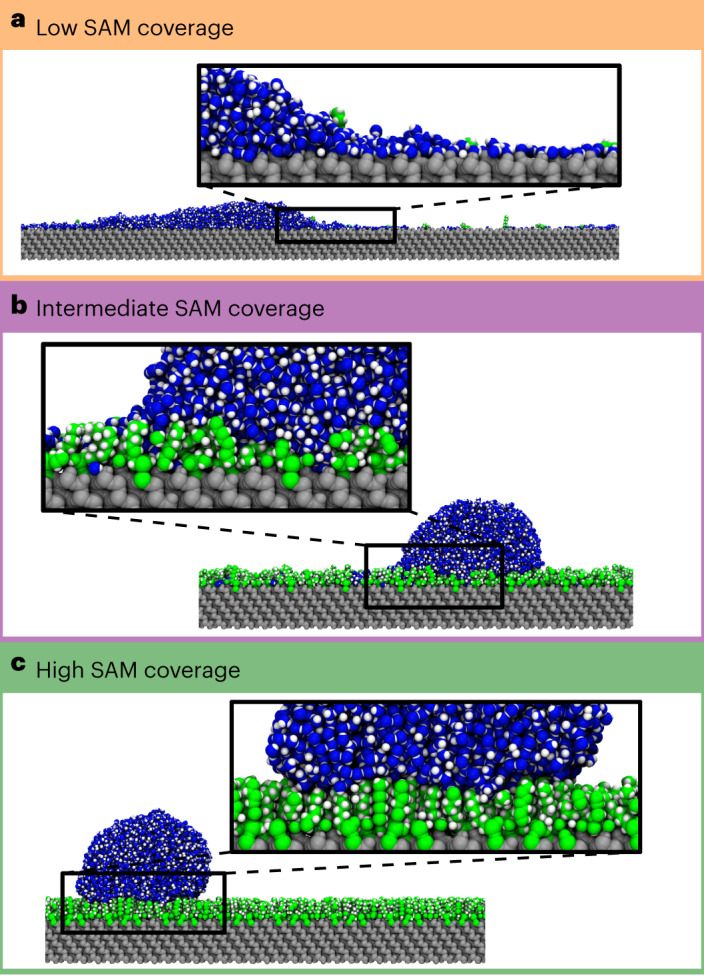
MD modelling showing the different mechanisms of the water droplet motion on SAM surfaces. **a**–**c**, A constant lateral force is applied to each water molecule to simulate droplet motion on low-coverage (0.1 molecules nm^−^^2^) (**a**), intermediate-coverage (2.0 molecules nm^−^^2^) (**b**) and high-coverage (3.4 molecules nm^−^^2^) (**c**) SAMs. On a low-coverage SAM, the hydrophilic SiO_2_ promotes the formation of an interfacial water film that acts as a lubricating layer for the droplet, resulting in low friction. On an intermediate-coverage SAM, the interfacial water on the SiO_2_ becomes patchy and less mobile, and instead of lubricating the droplet motion, the confined patches of interfacial water act as anchors for the droplet, resulting in higher friction. On a high-coverage SAM, liquid water permeates only marginally the SAM, and thus the droplet anchoring effect is reduced. Water adhesion to the hydrophobic alkyl tails is low, so the resulting friction is low.

The experimental results indicate that CLF depends on the molecular-scale heterogeneity of the SAM surface: most heterogeneous intermediate-coverage SAMs have higher friction than low- and high-coverage SAMs. However, it is not clear based on the experiments why we get these CLF values at each coverage regime, and what is the mechanism of friction in each case. For low-coverage SAMs, MD simulations show the presence of a thin, continuous film of interfacial water on the accessible SiO_2_ surface, extending from the droplet (Fig. 5a). The film is tightly adhered to the SiO_2_ surface due to hydrogen bonding between water molecules and the surface OH groups. According to residence time analysis (Supplementary Note 14), this interfacial water is not fully confined, as it can move within the interfacial layer or out from it due to thermal fluctuations. These fluctuations occasionally decrease energy barriers for reordering the hydrogen bonding network of molecules in the first layers of water, which allows easier propagation of water molecules at the contact line^37^. The interfacial water thus acts like a lubricating layer for the droplet, enabling the very low CLF observed experimentally. MD simulations show how the droplet moves on the interfacial water layer of the low-coverage SAM (Supplementary Note 15 and Supplementary Video 5).

Increasing SAM coverage decreases the accessible SiO_2_ surface, and thus the amount of interfacial water on it. According to the residence time analysis, this makes the interfacial water more confined, and it gradually loses its lubricating effect and starts hindering the molecular motion in the above water layers as the SAM coverage is increased. This is because intermediate-coverage SAMs have more OTS molecules that act as barriers restricting water molecule movement. The highest CLF is reached when interfacial water layer is in small, isolated patches and very confined, almost frozen, effectively acting as anchors for the droplet contact line (Fig. 5b and Supplementary Video 6). A further increase of SAM coverage reduces the areal density of these patches, reducing the CLF, and above 3 molecule nm^−^^2^ coverage the patches have practically disappeared (Fig. 5c and Supplementary Video 7). The residence time analysis shows that interfacial water on top of the SAM is very mobile due to the low adhesion between alkyl chains and water molecules.

It is interesting to note that MD simulations show that liquid water permeates only marginally through the OTS SAM above 3 molecules nm^−^^2^ (Fig. 5c and Supplementary Fig. 12). This finding (despite the scale differences in simulations) overlaps with the experimental finding that CAH and CLF stop decreasing beyond this coverage (Fig. 4). Interfacial water residence time above 3 molecules nm^−^^2^ is also constant within analysis accuracy. This would indicate that a SAM does not need to fully occupy the SiO_2_ surface and all its OH groups to minimize CAH and CLF: it is enough to block liquid water reaching the remaining small OH vacancies. Another interesting observation is that metal reactants, whose size is considerably larger than a water molecule, were still able to bond to the remaining OH vacancies of SAMs with coverage exceeding 3 molecules nm^−^^2^ (Fig. 3d), indicating that cavities of size much larger than a water molecule still exist in the SAM surfaces. We account for this by the difference in cohesive forces of polar water molecules and non-polar metal reactants: it is easier for the metal reactants to pass the non-polar alkyl tails than for polar water.

### Chemical heterogeneity and CLF of nanotextured surfaces

**Fig. 6:**
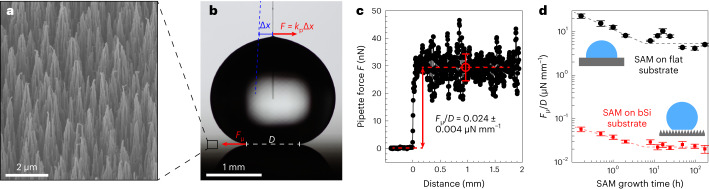
CLF on OTS SAM-coated nanostructured bSi surfaces. **a**, SEM image of the bSi surface coated with Al_2_O_3_ and SAM. **b**, Principle of the friction measurement with the MFS cantilever. Due to balance of forces, the deflection of the cantilever equals the droplet friction force while pulling the droplet on the surface. The velocity of the surface is 0.1 mm s^−1^. **c**, An example recorded CLF scan of a bSi surface coated with 48 h SAM surface recorded with the micropipette cantilever. The red dashed line at ca. 30 nN represents the regime (467 data points in this example) over which an average recorded friction force *F* of the droplet is calculated and the error bar represents correspondingly the standard deviation. **d**, Normalized CLF of SAM on flat (black circles) and bSi (red squares) substrates as function of SAM growth time. Data points represent average values obtained from multiple locations and with differently sized droplets (*n* = 5 for flat substrates and for bSi substrates *n* = 13, 17, 21, 13, 20, 24, 13, 19, 25, 16, 16; value reported in ascending order in terms of SAM growth time). Error bars of flat data series represent standard deviation of obtained normalized CLF. Error bars of rough data series represent 95% confidence interval of linear fit to the CLF data (Supplementary Note 16). Source data

So far, we have considered the relationship of chemical heterogeneity and CLF of flat surfaces. We want to extend our analysis to superhydrophobic surfaces that are rough in the nano/micrometre scale. The roughness facilitates an air layer below the droplet, called plastron, which substantially reduces solid-liquid contact between the droplet and the surface (that is, droplet is in the Cassie-Baxter state) resulting in very low CLF^38^. Here we varied the chemical heterogeneity of black silicon (bSi) surfaces with micrometre-sized spikes (Fig. 6a) by applying OTS SAM with varying growth time. Since SAM needs to have high enough ACA and RCA to support the Cassie-Baxter wetting state on the bSi structure, we applied an Al2O3 mid layer between the bSi and SAM (SAM grown on Al2O3 has wider range of CAH values than SAM grown on SiO2, such that both ACA and RCA are high; details in Supplementary Note 16).

SAMs grown on bSi surfaces yielded extremely superhydrophobic surfaces that are difficult to characterize accurately with CA goniometry^39^ or SA measurement due to extremely low droplet friction. Therefore, we used a more sensitive micropipette force sensor (MFS)^35^ for measuring the CLF. In MFS, CLF is measured by monitoring deflection of a thin cantilever while dragging a droplet along the surface (Fig. 6b and Supplementary Video 8), resulting in force curve as illustrated in Fig. 6c (detailed technique description is in Methods). The recorded normalized CLF (*F*_μ_/*D*) of 0.024 ± 0.004 μN mm^−1^ shown in Fig. 6c is extremely low. Based on equation ${F}_{{\upmu }}/D=(\rho {Vg}/D)\sin \alpha$, this friction value corresponds to a SA of just 0.01° for ca. 15 μl droplet. To the best of our knowledge, this is the most slippery surface ever reported. The CLF of SAM coated with flat Al_2_O_3_ was measured via CAs (Supplementary Note 16). Figure 6d shows how normalized CLF depends on the SAM growth time, which relates to SAM density, on each surface. Even though the normalized CLF of flat and rough surfaces differs by a factor of 300, the trend as a function of SAM deposition time is very similar. By increasing the SAM density (reducing surface chemical heterogeneity) the normalized CLF of smooth and rough surfaces drop by factors of 5 and 3, respectively (Supplementary Fig. 20). This indicates that surface chemical heterogeneity at molecular scales is an important factor for droplet friction not only on smooth surfaces with large solid-liquid contact area but also on rough, superhydrophobic surfaces with small solid-liquid contact area.

## Conclusions

We have explored how molecular-scale surface heterogeneity affects water CAH and CLF. We tuned the coverage of SAMs grown on a SiO_2_ surface, leading to surfaces with varying levels of molecular heterogeneity. Low CLF was observed for both low- and high-coverage SAMs with a regime of higher friction in between, which is in line with the level of heterogeneity in each of the coverage regimes. Yet, it is counterintuitive that hydrophilic, low-coverage SAM that has high adhesion to water can still yield low CLF. According to MD simulations, the interfacial water layer is not fully confined and acts as lubricant layer for the droplet, explaining the low CLF. When SAM coverage is increased, the layer becomes gradually more confined, and the lubricating effect switches to an anchoring effect, explaining the increased friction. At high SAM coverage, the anchoring effect disappears as the SAM becomes dense enough to block water from permeating through the SAM to the remaining surface OH vacancies. Even though high-coverage SAMs have cavities much larger than the size of a water molecule as demonstrated by reaction with metal reactants, water molecules pass only marginally through these cavities to contact the OH vacancies on the underlying hydrophilic substrate thereby keeping friction low. In conclusion, these findings shed new light on how molecular-level heterogeneity affects CAH and CLF, and thus help in engineering lower-friction surfaces for technologies from microfluidics to heat transfer and beyond.

## Methods

### Surface pre-treatment

Undoped, single-side-polished prime-grade silicon wafers with <100> orientation (University Wafer) were used as substrates for the experiments. The wafers were cut to smaller size with a diamond pen. The substrates were sonicated (P30H, Elmasonic) in ethanol (99.5 wt%, Etax AA, Altia Oyj) at approximately 30 °C temperature for 15 min at 37 kHz frequency to remove possible organic contamination and particle debris from the cutting step. Immediately after the sonication, Si substrates were rinsed under running flow of Milli-Q water for approximately 30 s followed by drying under N_2_ flow. The cleaned substrates were stored in polystyrene Petri dishes for maximum of 3 days. The cleaned Si substrates were inserted in UV-ozone cleaner (BioForce Nanosciences) for 30 min to remove any final organic residues from the surface and to introduce OH groups required for the chlorosilane adsorption on the surface. Immediately after the UV-ozone cleaning (within couple of minutes) the substrates were transferred to the ALD reactor for the OTS SAM growth step.

### SAM growth process

The OTS SAM growth was performed in an ALD reactor (Savannah S200, Veeco) equipped with pressure control unit for SAM dosing and viewports for ellipsometer measurement beam. The process is a modified version of similar process published earlier by Sundaram et al.^40^ The substrates were inserted in the reactor chamber pre-heated to 60 °C, after which the reactor was immediately pumped down to vacuum (base pressure of 7 Pa) and a 20 standard cubic centimetres per minute (SCCM) flow of N_2_ was applied through the reactor chamber. The substrates and reactor were let to stabilize for 30 min before starting the automated deposition process.

The deposition process started by a 15-ms pulse of water vapour through the reactor to provide a small amount of surface-bound water on the sample surface. The excess water vapour in the reactor atmosphere was purged off for 10 s with 20 SCCM N_2_ flow. Next, N_2_ flow was turned off and the valve between the reactor chamber and the vacuum pump was closed so that the reactor atmosphere became fully isolated. A liquid source of OTS (97%, Sigma-Aldrich) pre-heated to 65 °C was used to introduce a 50 ± 10 Pa dose of vapourized OTS into the reactor chamber. The OTS dose was kept in the chamber for the desired growth time, after which the valve between the reactor and the vacuum pump was opened to remove the non-adsorbed OTS and reaction by-products (HCl and H_2_O) from the chamber. For samples with growth times of 20 min and longer, the reactor atmosphere was replenished time to time by reconnecting the reactor to vacuum pump for 5 s, then after that disconnecting it from the vacuum and applying a fresh 50 ± 10 Pa dose of OTS to the chamber. The reason for the replenishment was to remove the generated reaction byproduct HCl and the air leaked into the reactor (reactor has inherent leakage rate of some Pa h^−1^). After the final dose, the reactor chamber was purged with 5 SCCM N_2_ flow for some minutes before venting. After removing the samples, they were stored in polystyrene petri dishes before characterization. Each characterization technique had its own allocated substrate per growth time to ensure the use of clean samples in each characterization.

### Preparation of bSi surfaces

The bSi surfaces were fabricated by a maskless cryogenic inductively coupled plasma (ICP) deep reactive ion etching (Oxford Plasmalab System 100, Oxford Instruments) process. The etching parameters were 7 min etching time, 1.3 Pa pressure, −110 °C temperature, forward power 6 W, ICP power 1,000 W and the gas flows were 18 SCCM O_2_ and 30 SCCM SF_6_. A 7 min cooling step was used after loading the wafer in the chamber to equilibrate the temperature.

Next, Al_2_O_3_ was grown on the bSi substrates with ALD^41^ in the Savannah S200 reactor. First, the bSi substrates were inserted to the reactor preheated to 60 °C, after which the reactor was immediately pumped down to vacuum. Along with bSi surfaces, reference smooth silicon substrates with similar pre-treatment as described above were inserted into the reactor as references. The substrates were let to stabilize 10 min. Next, ten cycles of trimethylaluminum (Volatec Oyj) + water process was performed for the substrates, resulting in about 1.0-nm-thick Al_2_O_3_ layer on the surfaces. The used pulse and purge times were 0.015 s and 30 s for both precursors, respectively. After this Al_2_O_3_ deposition step, OTS SAM was grown on the substrates similarly as described above.

### Deposition of labelling molecules for OH vacancy quantification

Organometallic compounds for labelling SAM surface OH vacancies were deposited with the Savannah S200 reactor. The used organometals are DEZ (99.9%, Volatec Oyj, Finland), TiCl_4_ (99.9%, Sigma-Aldrich), TDMAHf (99.99%, Sigma-Aldrich) and TTIP (99.999%, Sigma-Aldrich). The substrates with SAM were placed in the reactor chamber pre-heated to 150 ± 0.5 °C temperature, after which the reactor was immediately pumped down to vacuum. The samples were let to stabilize for 10 min inside the reactor, and the reactor N_2_ flow was increased to 20 SCCM for DEZ, TiCl_4_ and TTIP, and to 90 SCCM for TDMAHf for the rest of the process. After the stabilization period, the organometal molecules were pulsed in the reactor (15 ms, 100 ms, 200 ms and 100 ms for DEZ, TiCl_4_, TDMAHf and TTIP, respectively), followed by a 60-s purge. This pulse + purge was performed ten times to ensure that all the accessible OH vacancies got filled with marker molecules. No water pulses were performed to prevent multilayer growth of metal oxides. After the last purge, the reactor was vented, and the samples were removed from the reactor and stored in polystyrene Petri dishes until the characterization.

### Operando ellipsometry

Operando ellipsometry measurements were performed with a spectroscopic ellipsometer (M2000UI, JA Woollam) through viewports of the Savannah S200 reactor chamber. The recording was performed at 70° angle from surface normal with acquisition time of 4 s used for SAMs grown 12 h or less and 10 s for SAMs grown longer. The ‘High accuracy mode’ of the device measurement software (CompleteEASE ver. 6.51, JA Woollam) was used during the acquisition. The same software was used for fitting an optical model for obtaining the SAM thickness. The model is composed of three layers: the bottom layer represents the crystalline silicon substrate, the middle layer represents the native oxide of the silicon wafer, and the top layer represents the SAM layer. This model was used for estimating SAM thickness, refractive index and coverage (that is, areal density of OTS SAM molecules). More detailed description of ellipsometry is in Supplementary Note 3.

### FTIR

FTIR were performed with an attenuated total reflection (ATR) accessory (VariGATR, Harrick Scientific) installed in an infrared spectrometer (Tensor 27, Bruker) equipped with a mercury cadmium telluride (MCT) detector. The measurements were performed adapting the protocol published by Lummerstorfer et al.^42^ The Ge crystal of the ATR unit was cleaned before each background or sample measurements first with 2-butanone (99.5%, Merck) and then 30 min in the UV-ozone cleaner to remove any organic residues. After mounting the Ge crystal back to the ATR unit, the unit was purged for 15 min with dry N_2_ flow to remove moisture. First, the background spectrum was recorded against a cleaned, uncoated Si substrate. After re-cleaning the crystal, the sample was measured. The background and sample were measured by pressing the substrate top surface with 600 ± 20 N force against the germanium crystal, irradiating with *p*-polarized measurement beam at 60° angle against the Ge surface normal, and recording the spectra 1,024 times between 2,700 cm^−1^ and 3,100 cm^−1^ with 2 cm^−1^ resolution. The obtained spectra were corrected for horizontal baseline, after which the peak locations were obtained using the Bruker OPUS (ver. 7.2) software function for finding the peak locations (half width at half maximum).

### AFM

The AFM measurements were performed in ambient environment using Dimension Icon AFM (Bruker AXS; formerly Veeco) with a ScanAsyst-air cantilever (sharp silicon nitride tip with a nominal radius of 2 nm for PeakForce Tapping in the air). The scan size was set to 1 μm × 1 μm with 512 pixel × 512 pixel resolution, and the scanning was done with a scan rate of 1 kHz. ScanAsyst Auto control was set to ‘individual’ for the samples with PeakForce Amplitude of 50 nm. Spring constant and PeakForce frequencies were 0.4 Nm^−1^ and 2 kHz, respectively, for all samples.

### XPS

The XPS measurements were made using Kratos Axis Ultra system (Kratos Analytical), equipped with a monochromatic AlKα X-ray source. All measurements were performed with 0.3 mm × 0.7 mm analysis area. High-resolution scans were performed with 20 eV pass energy with 0.1 eV step size. The spectra were obtained from three different locations for each sample.

### RBS

RBS spectrometry was performed using a 1.6 MeV ^4^He^+^ ion beam. The backscattered He is detected by a ring of 14 individual Si (PIPS) detectors, each at a scattering angle of 160°. The samples were kept perpendicular to the incident beam, making the scattering geometry for each detector identical and thus allowing the spectra to be summed. The combined solid angle is high, approximately 70 msr. The beam spot on the sample was 3 × 3 mm^2^.

The dose (number of incident ions) is normalized by means of detection of backscattering from a rotating vane beam chopper. The calibration of chopper yield to number of ions was performed by measuring an RBS spectrum from a thick stoichiometric SiO_2_ film and obtaining the solid angle and dose product from by fitting a simulation to the measurement. Additionally, a thin film sample (few nm Au) on SiO_2_ was measured every time the samples were changed, allowing the monitoring of detector energy calibration drift and stability of the chopper yield calibration. When measurements were split over several days, some samples were re-measured to study the repeatability of the results and role of beam induced changes, that is, possible Hf loss via sputtering. Repeated measurements were found to produce identical results within statistical uncertainty.

The Hf areal density is calculated from number of counts in the Hf peak in the spectrum by assuming Hf is at the sample surface and using an appropriate scattering cross-section. Background in the spectra is negligible even when Hf areal density is 1 at. nm^−^^2^ (10^14^ at. cm^−^^2^) and no background reduction was performed. Statistical uncertainty can be calculated from the number of Hf counts and number of chopper counts and is between 1% and 2% in these measurements. In addition to the statistical uncertainty there is some minor systematic uncertainty due to the chopper yield calibration process.

### Wetting characterization

Milli-Q water was used as probe liquid in all wetting characterization.

#### ACA and RCA measurements

ACAs and RCAs were recorded using an optical tensiometer (Attension Theta, Biolin Scientific) and OneAttension software (ver. 3.2, Biolin Scientific). ACA and RCA were obtained by adapting the protocol published by Huhtamäki et al.^43^. Detailed description is in Supplementary Note 17 and Supplementary Fig. 21. The CAs were obtained from three different locations on each sample.

#### SA measurements

SAs were recorded with the same optical tensiometer as ACA and RCA. SA was measured by first depositing a sessile droplet on a horizontal sample surface (accuracy of level ± 0.1°). The sample stage was then started to tilt at a constant tilt rate. There is a trade-off between measurement accuracy and droplet evaporation regarding the tilt rate, so for surfaces with small SA a tilt rate of 5° min^−1^ was used, and for experiments with larger SA of 15° min^−1^ was used to limit the droplet evaporation. The droplet was considered to slide when both its advancing and receding fronts were moving. At the starting point of the slide, droplet baseline width, volume and tilt angle were recorded. The experiments were recorded with droplet sizes of 5 μl, 10 μl and 20 μl, each at three different locations.

#### Droplet friction with MFS

The MFS technique was used to directly measure the droplet friction on the very slippery SAM-coated bSi samples using a previously developed protocol^35,44^. A micropipette was manufactured from thick glass capillaries (inner diameter/outer diameter 0.75/1 mm, World Precision Instruments, model no. TW100-6) using a micropipette puller (Narishige, model no. PN-31) and cut with a microforge (Narishige, model no. MF-900) to a cantilever length of 2.5 cm. The straight cantilever was force calibrated (*k*_*p*_ = 5.6 ± 0.1 μN mm^−1^) by mounting it horizontally and pushing out a small water drop that acted as a control weight and then measuring deflection of the cantilever (detailed description of force calibration by Backholm et al.^44^). To measure the sliding friction of the different samples, the cantilever was mounted vertically above the surface and water drop was pushed out through the micropipette (Fig. 6b). By moving the substrate with a motor in the *y* direction (out of the page in Fig. 6b), the drop-micropipette system reaches its equilibrium *x* position (left–right direction in Fig. 6b). A side-view camera (Canon 80D capturing at 30 fps using a Canon MP-E macro lens at ~3× magnification) was then started to capture the equilibrium (*F* = 0 N position) and 3–4 s later the motor started moving the substrate below the drop in the x-direction at a constant speed of *v* = 0.1 mm s^−1^ (see example Supplementary Video 8). The drop, attached to the micropipette by capillary force *s*, was dragged over the surface and the micropipette deflection was analysed from the video using MATLAB^40^, rendering a force–time graph as shown in Fig. 6c. In this velocity regime (0.1 mm s^−1^) the resistive forces of droplet internal motion dissipation are small^45^. The contact region diameter (*D*) was measured from the same video at the start and end of the sliding friction regime. These experiments were repeated with drops of different sizes (drop radii in the range of *R* = 0.7–1.45 mm) to get a robust estimate of the normalized CLF, *F*_μ_/*D*. To minimize noise in the force data, the whole setup was built on an active antivibration table (Halcyonics i4large, Accurion) resting on a sturdy optical table (Thorlabs). The entire setup was finally shielded with a closed cardboard box during the experiments to avoid micropipette vibrations due to air flow.

### SEM

Scanning electron microscopy (SEM) image of the bSi surface was obtained with Sigma VP (Zeiss). Before imaging, a 5 nm AuPd film was sputtered (Leica EM ACE600) on top of the bSi surface to reduce charging of the surface during imaging. The surface was tilted to 45° angle, and the image was captured using in-lens detector, 2.00 kV acceleration voltage and with working distance of 4.7 mm resulting in 10,000× magnification.

### MD simulations

We use the LAMMPS MD engine^46,47^ to perform the simulations. For the silica substrate we used the Emami et al. silica force field^48^ and for the OTS chain we used the force field from Roscioni et al.^49^ and Castillo et al.^50^ The flexible CVFF^51^ model was used for water. Final equilibration runs were done at 300 K, using the Nosé–Hoover thermostat^52–54^ with a 0.1 ps damping factor. Electrostatics were calculated with the particle–particle–particle–mesh (P3M) method^55^. Analysis was done with the MDAnalysis python library^56^. The timestep was 1 fs. Atom colouring in all figures is illustrated as follows: SiO_2_ substrate hydrogen, oxygen and silicon in grey; OTS carbon, oxygen and silicon in green, and hydrogen in white; water hydrogen in white, and oxygen in blue. Further details of the simulations are in Supplementary Notes 7, 11, 15 and 18.

## Online content

Any methods, additional references, Nature Portfolio reporting summaries, source data, extended data, supplementary information, acknowledgements, peer review information; details of author contributions and competing interests; and statements of data and code availability are available at 10.1038/s41557-023-01346-3.

### Supplementary information

Supplementary Information Supplementary Notes 1–18, Figs. 1–21 and Tables 1–3 and captions of Supplementary Videos 1–8.

Supplementary Video 1 **Thermal motion of OTS SAM at room temperature**. The video represents SAM surface with 0.77 molecule nm^−^^2^ coverage over a time period of 10 ns.

Supplementary Video 2 **SA experiment of 10** **μl droplet on OTS SAM with 0.12 molecules** **nm**^**−**^^**2**^ **coverage**. The camera tilts along the sample, so sample surface does not tilt visibly. The playback speed is 5× real time.

Supplementary Video 3 **SA experiment of 10** **μl droplet on OTS SAM with 0.50 molecules** **nm**^**−**^^**2**^ **coverage**. The camera tilts along the sample, so sample surface does not tilt visibly. The playback speed is 5× real time.

Supplementary Video 4 **SA experiment of 10** **μl droplet on OTS SAM with 3.2 molecules** **nm**^**−**^^**2**^ **coverage**. The camera tilts along the sample, so sample surface does not tilt visibly. The playback speed is 5× real time.

Supplementary Video 5 **MD simulation of droplet sliding on OTS SAM with 0.25 molecules** **nm**^**−**^^**2**^ **coverage**. The video shows a droplet with 3,493 water molecules moving on the OTS SAM surface over a time period of 5.0 ns.

Supplementary Video 6 **MD simulation of droplet sliding on OTS SAM with 0.77 molecules** **nm**^**−**^^**2**^ **coverage**. The Video shows a droplet with 3,493 water molecules moving on the OTS SAM surface over a time period of 5.0 ns.

Supplementary Video 7 **MD simulation of droplet sliding on OTS SAM with 3.44 molecules** **nm**^**−**^^**2**^ **coverage**. The video shows a droplet with 3,493 water molecules moving on the OTS SAM surface over a time period of 1.2 ns.

Supplementary Video 8 **Example of MFS scan of SAM-coated bSi surface**. The droplet volume is ca. 10 µl and total scan distance approximately 2 mm. In the video, droplet and camera are kept stationary while the surface is moved left.

### Source data

Source Data Fig. 2 Numerical data shown in Fig. 2a–c and Fig. 2k.

Source Data Fig. 3 Numerical data shown in Fig. 3c,d.

Source Data Fig. 4 Numerical data shown in Fig. 4a,c.

Source Data Fig. 6 Numerical data shown in Fig. 6c,d.

Source Data Extended Data Fig./Table 1 Numerical data shown in Extended Data Fig. 1b,d,e.

## Data Availability

The data supporting the findings of this study are available within the paper and its Supplementary Information. Source data are provided with this paper.
