# Publisher Correction: Droplet slipperiness despite surface heterogeneity at molecular scale

**DOI:** 10.1038/s41557-023-01401-z

**Published:** 2023-11-28

**Authors:** Sakari Lepikko, Ygor Morais Jaques, Muhammad Junaid, Matilda Backholm, Jouko Lahtinen, Jaakko Julin, Ville Jokinen, Timo Sajavaara, Maria Sammalkorpi, Adam S. Foster, Robin H. A. Ras

**Affiliations:** 1 Department of Applied Physics, Aalto University, Espoo, Finland; 2 Centre of Excellence in Life-Inspired Hybrid Materials (LIBER), Aalto University, Espoo, Finland; 3 Department of Chemistry and Materials Science, Aalto University, Espoo, Finland; 4 Department of Physics, University of Jyväskylä, Jyväskylä, Finland; 5 Department of Bioproducts and Biosystems, Aalto University, Espoo, Finland; 6 Nano Life Science Institute (WPI-NanoLSI), Kanazawa University, Kakuma-machi, Kanazawa, Japan

**Keywords:** Surface chemistry, Wetting

Correction to: *Nature Chemistry* 10.1038/s41557-023-01346-3, published online 23 October 2023.

In the version of the article initially published, there was an error in Fig. 6a, in which the scale bar, “2 µm” incorrectly appeared as “2 mm”; and in Fig. 6b, the equation *F = k*_p_∆*x* was incorrectly shown as F = K∆x. These errors have been corrected in the HTML and PDF versions of the article.

