# Supplementary material for: Droplet slipperiness despite surface heterogeneity at molecular scale
Source: Nat Chem. 2023 Oct 23;16(4):506–13. doi: 10.1038/s41557-023-01346-3 (PMC10997520; doi:10.1038/s41557-023-01346-3)
Supplement: Supplementary file 1 — Supplementary Notes 1–18, Figs. 1–21 and Tables 1–3 and captions of Supplementary Videos 1–8. [file 41557_2023_1346_MOESM1_ESM.pdf]

nature chemistry

Article https://doi.org/10.1038/s41557-023-01346-3

Droplet slipperiness despite surface heterogeneity at molecular scale

In the format provided by the authors and unedited

Supplementary information

Table of contents

Supplementary Note 1: Ideality of the monolayer growth 2
Supplementary Note 2: Detailed analysis of OTS SAM growth process 3
Supplementary Note 3: Determination of OTS SAM film thickness, refractive index, and coverage from ellipsometer data. 7
Supplementary Note 4: Monolayer growth model 9
Supplementary Note 5: Si substrate surface stability inside the ALD reactor 10
Supplementary Note 6. Fourier transform infrared spectroscopy (FTIR) of SAMs 12
Supplementary Note 7: Alkyl chain tilt angle and OTS molecule height of simulated SAM surfaces 13
Supplementary Note 8: Labelling OH vacancies with metal reactants 14
Supplementary Note 9: Details of XPS analysis 16
Supplementary Note 10: Estimation of accessible OH groups from RBS analysis 17
Supplementary Note 11: MD simulations of droplets on SAM surfaces 18
Supplementary Note 12: Prediction of SAM contact angle with Cassie's law 21
Supplementary Note 13: Sliding angle measurements 22
Supplementary Note 14: Mobility of interfacial water molecules via residence time analysis 24
Supplementary Note 15: MD simulation of droplet sliding 26
Supplementary Note 16. Comparison of contact line friction of SAM on smooth and bSi surfaces 27
Supplementary Note 17: Determination of advancing and receding contact angles 30
Supplementary Note 18: Details of MD simulation of OTS SAM assembly on SiO_2 31
Captions of Supplementary Videos 32
Supplementary Information references 33

Supplementary Note 1: Ideality of the monolayer growth

Trichlorosilane molecules can form either smooth self-assembled monolayers via self-limiting adsorption on the substrate surface (as illustrated in the main text Fig. 1) or rougher films where silane molecules react with each other in a process termed polymerization, as has been presented earlier by Fadeev *et al.*[1] Water has a complex role in trichlorosilane chemistry because it is a critical reactant in the hydrolysis of trichlorosilanes and it is also a reaction product of the condensation and polymerization reactions. The self-limiting growth type requires a dry reaction environment, whereas the polymerization growth type occurs when the environment contains water. When the reaction environment is not sufficiently dry, the overall growth can be a mixture of these two growth types. In this supplementary note we discuss the aspects that support the smooth monolayer type of growth over the growth type involving polymerization.

In this work, the growth of OTS films is performed using an atomic layer deposition reactor capable of providing excellent control on minimizing the water content in the reactor atmosphere by having the reactor in vacuum, purging it with nitrogen gas and having it at temperature of 60 °C (reactor is always kept at least at 60 °C to avoid accumulation of water in the reactor walls prior to the OTS growth process). The reactor has a base pressure of ca. 7 Pa, which is mostly due to leakages of normal air that has ca. 1% water content. The OTS pulse pressure is 50 ± 10 Pa, which is much higher than the partial pressure of residual water in the reactor atmosphere. Therefore, OTS molecules in vapor phase are unlikely to hydrolyze and polymerize with other OTS molecules in the reactor atmosphere.

The OH-group rich SiO_2 substrate surface is hygroscopic and may have a thin film of surface-bound water despite the otherwise dry reaction environment. Therefore, the hydrolysis of the OTS molecules likely occurs only at or in the vicinity of the substrate surface, and due to the proximity of the surface OH groups, the hydrolyzed OTS molecules are also likely to bond to the surface. Once a first Si-O-Si bond between an OTS molecule and a surface is formed, it is likely that the remaining two Si-OH moieties of the hydrolyzed OTS molecule will also bond to the substrate surface or possibly to a neighboring OTS molecule already adsorbed to the surface if it still has a remaining Si-OH moiety. Therefore, OTS should not substantially polymerize vertically with other OTS molecules while bonding to the substrate is the more probable scenario.

Experimental evidence also suggests growth of smooth self-assembled monolayers without substantial vertical polymerization. Firstly, the formed OTS films maintain the smoothness of the underlaying silicon wafer substrate according to atomic force microscopy measurements (see Figure 2d-g in the main text). Vertical polymerization would lead to increased RMS roughness, which we do not observe at any OTS SAM coverage regime. Secondly, the coverage increase follows a standard monolayer growth model (Supplementary Note 4) that assumes growth rate being proportional to the fraction of unoccupied surface area. This indicates that OTS molecules do not grow on top of other OTS molecules that have bound earlier to the surface, i.e., the growth is clearly self-limiting. Lastly, ellipsometry shows that the growth of OTS film also saturates to thickness that is nearly equal to the length of a single OTS molecule. For the above reasons, the OTS film can be assumed to form as a smooth monolayer with negligible amount of vertical polymerization.

Supplementary Note 2: Detailed analysis of OTS SAM growth process

Covalent bonding of OTS molecules to SiO_2 surfaces releases HCl as by-product. At the same time, the partial pressure of OTS vapor decreases in the reactor slowing down the growth rate. Furthermore, over time air leaks into the ALD reactor gradually bringing in moisture and other airborne impurities. For these reasons, the reactor was replenished periodically for the OTS SAM depositions of 20 min and longer by reconnecting the reactor to vacuum pump and removing excess OTS and reaction byproduct HCl and applying a new dose of OTS. As the growth of OTS SAM is fast in the beginning, the first exposure step was kept short, maximum 15 min. The next 1-4 steps were slightly longer, up to 27.5 min, and the last steps after that were each 60 min. Table 1 shows list of exposures used for each prepared SAM surface.

Table 1 | Prepared OTS SAM samples. Count and length of each replenishment steps are shown in the columns of the table.

Growth time	Exposures: count x duration per exposure (min)			Total count
	Short	Intermediate	Long	
**30 s**	1 x 0.5			1
**1 min**	1 x 1			1
**2 min**	1 x 2			1
**5 min**	1 x 5			1
**8 min**	1 x 8			1
**10 min**	1 x 10			1
**12 min**	1 x 12			1
**15 min**	1 x 15			1
**20 min**	1 x 10	1 x 10		2
**30 min**	1 x 10	1 x 20		2
**1 h**	1 x 10	2 x 25		3
**2 h**	1 x 10	4 x 27.5		5
**4 h**	1 x 10	4 x 27.5	2 x 60	7
**8 h**	1 x 10	4 x 27.5	6 x 60	11
**12 h**	1 x 10	4 x 27.5	10 x 60	14
**18 h**	1 x 10	4 x 27.5	16 x 60	21
**24 h**	1 x 10	4 x 27.5	22 x 60	27
**48 h**	1 x 10	4 x 27.5	46 x 60	51
**96 h**	1 x 10	4 x 27.5	94 x 60	99
**168 h**	1 x 10	4 x 27.5	166 x 60	171

The SAM growth was monitored *in-situ* with *operando* ellipsometry, i.e., monitoring the SAM growth occurring in the ALD reactor chamber in real time. Fig. 1a-e shows the SAM thickness development of each prepared SAM surface. In each case, the growth starts by a rapid increase of thickness (marked with first * in the main text Fig. 2a). This can be accounted for rapid physisorption of OTS molecules onto the substrate surface. The physisorbed OTS molecules start soon covalently bonding to the substrate OH groups, releasing HCl by-product. This mass loss causes a step decrease in the observed film thickness. After roughly 1-2 minute of growth, observed thickness starts again to increase as rate of HCl removal decreases below OTS adsorption rate.

Once first exposure is finished, the ALD reactor is reconnected to vacuum pump for 5 s, which removes most of produced HCl and also excess OTS from the reactor. This effectively removes physisorbed OTS from the sample surface, which causes a rapid yet small decrease of observed film thickness, and the remaining

thickness represents covalently adsorbed SAM. For those runs with multiple exposures, a new OTS dose is introduced into the ALD reactor chamber (marked with second * in the main text Fig. 2a), which causes a similar peak in the observed thickness as with the first exposure. Again, more molecules get physisorbed onto the substrate surface, and the molecules start binding covalently to the substrate over time before the next reactor purge that removes all the physisorbed molecules. Each following OTS exposure cycle behaves similarly, however the amount of physisorption gets smaller as OTS coverage increases, which appears as reduction of thickness decrease right after the dosing.

Fig. 1f shows the decrease of observed thickness after the last purge for each SAM growth. This thickness decrease is due to the removal of physisorbed molecules like discussed above. The decrease gets smaller towards the longer runs, and after 24 h deposition no removal is observed, which coincides with the slowing of the growth. The introduction of first replenishment event (second OTS dose) increases the thickness decrease after the deposition. Therefore, it is likely that replenishment cycles increase the growth rate, affecting the total growth slightly (i.e., 20 min growth would have had lower final thickness had it been performed with one OTS dose only). After the thickness decrease, the remaining OTS is covalently bonded, as introduction of ca. 100 Pa and 1 s water vapor pulse into the reactor does not cause observable changes in the SAM thickness, see Fig. 2. Had there been substantial amounts of unhydrolyzed physisorbed OTS on the surface, the hydrolysis would have likely caused an observable change in ellipsometric SAM thickness.

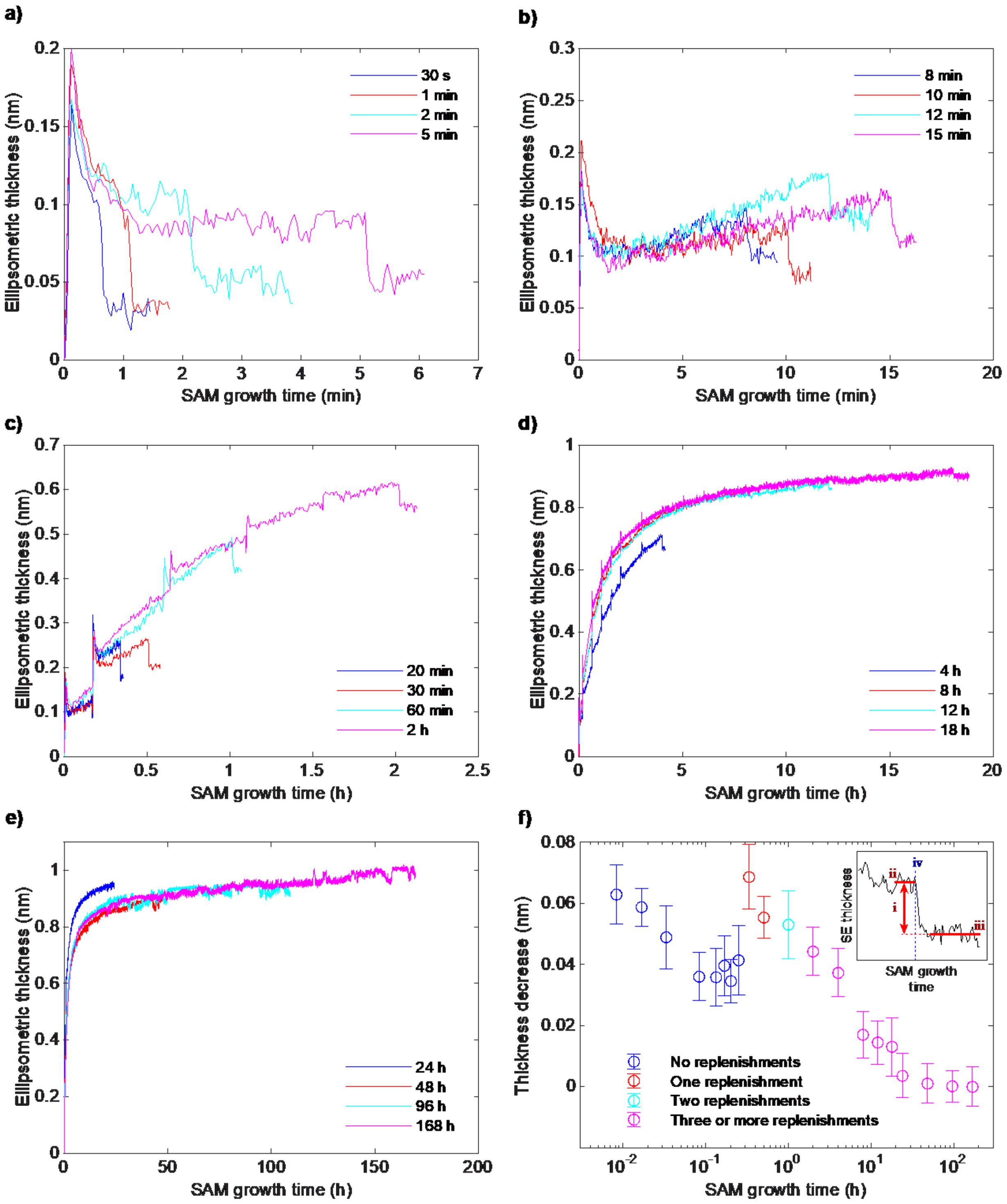

Fig. 1 | Monitoring OTS SAM growth with ellipsometer. a-e) SAM ellipsometric thickness growth of all depositions with total time ranging from 30 s to 168 h. **f)** Magnitude of thickness decrease at the end of each deposition. The inset shows how thickness decrease (i) is calculated by taking the difference of average thickness (ii) before and (iii) after the final purge (iv) final purge. Inset data from 2 min deposition. Different colors show how many replenishment (= evacuation + dose) steps there have been in each deposition process, and error bars represent pooled standard deviation of averaged thickness regions before and after the final purge.

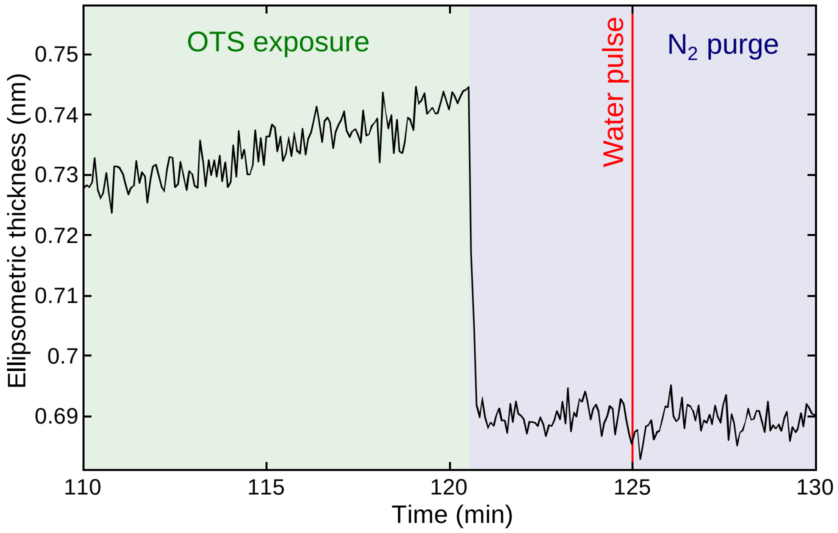

Fig. 2 | Effect of water vapor on the OTS SAM thickness after final purge. The green shaded region represents the time when reactor contains OTS dose. The blue shaded region represents the time when reactor is being purged with N_2. The red line represents the moment when water vapor pulse was introduced into the reactor.

Supplementary Note 3: Determination of OTS SAM film thickness, refractive index, and coverage from ellipsometer data.

Ellipsometry can be used to obtain SAM thickness and refractive index by fitting an optical model describing the surface structure to the measured Ψ and Δ values. In the case of OTS SAM deposited on Si wafer with native oxide layer, the optical model composes of three layers. The bottom layer represents the crystalline Si substrate, and it is modeled with tabulated values for refractive index. The middle layer represents native oxide of the silicon wafer. Its thickness is set to 1.5 nm (see below for details) and its refractive index is modeled with Cauchy dispersion equation $n(\lambda) = A + \frac{B}{\lambda^2} + \frac{C}{\lambda^4}$, where λ is the wavelength of light and A, B, and C are constants that are fitted from data recorded just before the start of SAM growth. The top layer represents the OTS SAM. To estimate the average SAM thickness with the model, refractive index was fixed to 1.45[2], and to estimate the refractive index, thickness was fixed to 1.0 nm, which corresponds to OTS SAM maximum height obtained in the MD simulations in this work. As the SAM thickness is in the order of 0.1 – 1.0 nm, ellipsometry is not sensitive enough so that both thickness and refractive index of the SAM could be fitted simultaneously.

The exact thickness of the native oxide is difficult to estimate with sub-Ångström accuracy. Therefore, we tested how sensitive our analysis for SAM thickness is for the set native oxide thickness. Fig. 3a shows the obtained SAM thickness for three different native oxide thickness values used in the analysis. The SAM thickness is within the error margins for each case, meaning the analysis is not sensitive for the set value of the native oxide thickness (in range from 1 nm to 2 nm).

The recorded SAM thickness and refractive index are dependent on the assumed value of the other parameters. For example, Fig. 3b shows how thickness of a 4 h SAM is different depending on what refractive index value is used in fitting of the optical model to the ellipsometer data. This leads to a systematic error if there is a difference between the assumed and real refractive indices, meaning that the absolute obtained ellipsometer values may have an inaccuracy of ca. 10% but the relative difference between the samples is still accurate.

The obtained refractive index can be further transformed into SAM coverage, i.e., areal density of OTS molecules[1,2]. In short, mass uptake per unit area of the film can be calculated using Equation 1

$$\Delta m = \frac{Md}{A_{\mathrm{MR}}}\left(\frac{n^2 - 1}{n^2 + 2}\right) \tag{1}$$

where M and A_{MR} are molecular mass and molar refractivity of the SAM, respectively, d is SAM thickness and *n* is refractive index of the SAM. Areal density of molecules adsorbed on the surface is obtained by dividing both sides of Equation 1 with mass of a single molecule, yielding Equation 2

$$\frac{\Delta m}{m_{\mathrm{molecule}}} = \rho_{\mathrm{areal}} = \frac{Md}{A_{\mathrm{MR}} m_{\mathrm{molecule}}}\left(\frac{n^2 - 1}{n^2 + 2}\right) \tag{2}$$

In the case of OTS, M = 189, A_{MR} = 46.7 (values obtained using principle explained by Cuypers *et al.*[3]), m_{molecule} = 3.14 $\cdot$ 10^{-25} kg and d = 1.0 nm as assumed during determination of refractive index from the ellipsometer data. Again, it must be noted that calculation of molecule areal density is sensitive to the assumption of SAM thickness. If thickness is assumed to be 0.9 nm or 1.1 nm instead of 1.0 nm, the coverage shifts ± 10%, see Fig. 3c.

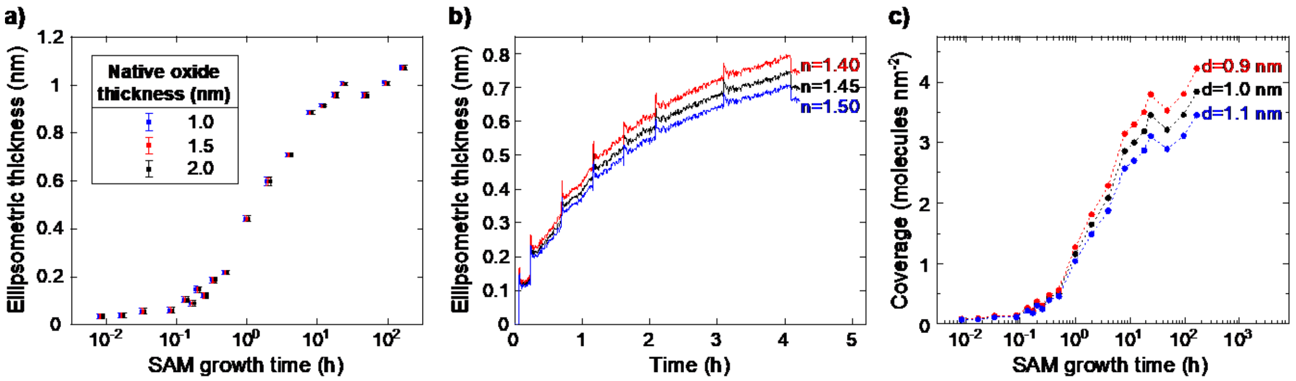

Fig. 3 | Ellipsometer accuracy. a) SAM final ellipsometric thickness with different values used for native oxide thickness. The 1.0 nm and 2.0 nm data series are shifted in x direction for better visibility. Error bars represent standard deviation of average SAM ellipsometric thickness after the final purge due to signal noise. **b)** Ellipsometric thickness recorded during 4 h OTS SAM growth with three different refractive index values used in the model fitting. **c)** SAM coverage of each sample for three different thickness values used in model fitting and coverage calculation.

Supplementary Note 4: Monolayer growth model

The growth of the SAM can be modeled with a simple growth model[4]. It can be assumed that growth rate is proportional to coverage σ, and there is no desorption (as molecules eventually bond covalently to the surface) leading to Equation 3

$$\frac{d\sigma}{dt} = k(1-\sigma) \tag{3}$$

where k is chemical reaction specific rate parameter. Solving for coverage yields Equation 4

$$\sigma = \sigma_{\max}(1 - e^{-\frac{t}{\tau}}) \tag{4}$$

where $\sigma_{\max}$ is the maximum achievable coverage and τ is the growth half time.

Supplementary Note 5: Si substrate surface stability inside the ALD reactor

The substrate stability inside the ALD reactor is essential when growing SAMs for several days. We verified the surface stability by keeping a substrate inside an idle ALD reactor over 72 h time period. First, a Si substrate was pre-treated similarly as described in the Methods section, and then inserted into the ALD reactor pre-heated to 60 °C. Next, the reactor was pumped down to vacuum and a 20 sccm N_2 flow was added for 72 h. The sample surface was monitored with *operando* ellipsometry during that time. The sample was modelled with two-layer model: the first layer is the crystalline Si substrate (fixed values for refractive index) and the second layer is the native oxide that is modelled with Cauchy dispersion. In the beginning of the experiment, the thickness of the native oxide was set to 1.50 nm, and Cauchy parameters A, B, and C were fitted to obtain the refractive index of the native oxide. Then the Cauchy parameters were fixed to the fitted values, and thickness of the native oxide was fitted during the *in-situ* monitoring of the sample.

The results are shown in Fig. 4 below. The detected changes are very small and random. This indicates that the native oxide thickness remains stable inside the ALD reactor, and it is valid to assume a constant native oxide thickness during SAM growth monitoring. We note also that the fit of native oxide thickness is sensitive for any changes at its surface, in particular if the amount of surface bound water would change. Therefore, the amount of surface bound water is also very stable, which is important for the SAM growth stability.

After the 72 h waiting period, another, freshly pre-treated Si substrate was inserted into the ALD reactor. Next, a normal OTS SAM deposition was performed for the two substrates inside the ALD, after which advancing and receding contact angles of both samples were measured. The results are in Table 2, and they show that SAM grew very similarly on both samples. This indicates that surface OH group density has also remained very constant during the 72 h wait period inside the ALD reactor, which is important for the late stage SAM growth and quantification of OH groups with the metal reactants that occurred after the SAM growth.

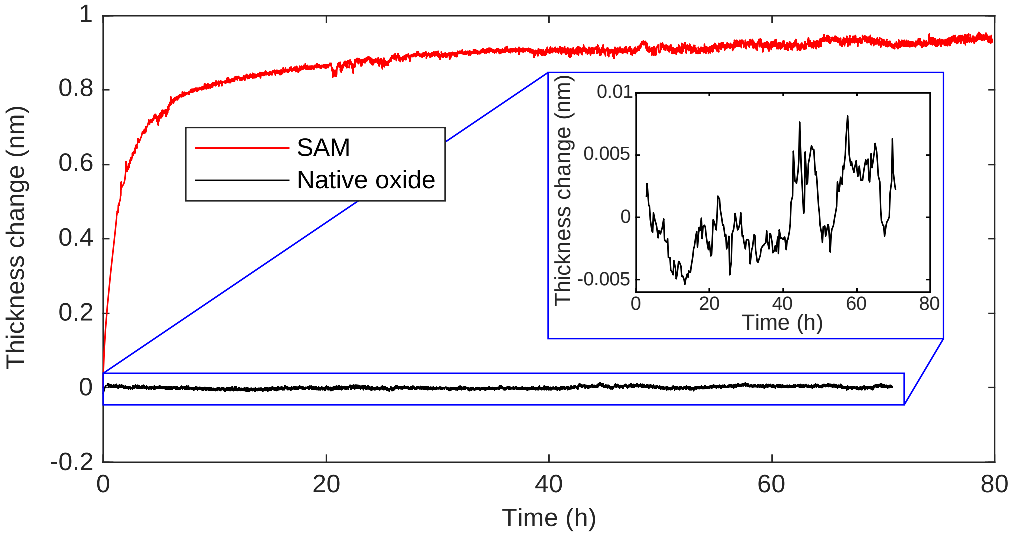

Fig. 4 | Stability of native oxide. Comparison of thickness change of native oxide and SAM over 72 h period. Inset shows a zoom-in to the measured thickness variations of the native oxide. The inset data is smoothed with running average to better visualize the long-term changes in the detected thickness.

Table 2 | Advancing and receding contact angles of OTS SAM grown on UV-O_3 activated surfaces with 72 h aging and no aging prior to SAM growth. Contact angle error margin is ±0.7° for each measurement and is based on standard deviation of mean angles detected from three different surface locations.

Surface	Freshly activated	72 h aged
**Contact angles**	103.5°/96.8°	103.1°/96.1°

Supplementary Note 6. Fourier transform infrared spectroscopy (FTIR) of SAMs

Level of SAM crystallinity can be determined with FTIR as the peak position of the asymmetric CH_2 stretching band depends on the alkyl tail configuration[5,6]. For alkyl tails in all-trans configuration the peak occurs near 2917 cm^{-1}, and the more cis conformers the tail has the more the peak is blue shifted, i.e., wavenumber increases. Fig. 5 shows FTIR spectra obtained for five SAMs with different growth times. The asymmetric CH_2 peak occurs redshifts from 2929 cm^{-1} for 2 min growth time to 2926.5 cm^{-1} for 168 h growth time. This indicates a small straightening of alkyl tails during the SAM growth, but no crystallization within the explored growth time range.

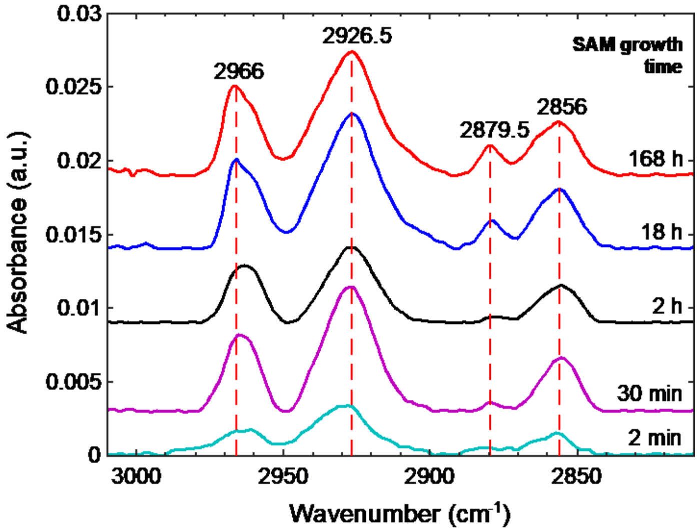

Fig. 5 | Fourier transform infrared spectroscopy of prepared SAM surfaces. The red dashed lines show peak locations of 168 h OTS SAM graph as guide for eye for comparison between spectra. The curves are background subtracted and leveled spectra with baseline shift for 30 min – 168 h SAM spectra.

Supplementary Note 7: Alkyl chain tilt angle and OTS molecule height of simulated SAM surfaces

We extended the MD simulations of the generated SAMs (Supplementary Note 18) by further 42 ns. The last 40 ns were used for analyzing the average tilt angle of the alkyl chains of the OTS molecules. (See Video 1 how alkyl tails wiggle due to thermal motion.) For that, we calculated the angle β as depicted on Fig. 6. The vector following the alkyl chain goes from the silane oxygen bonded to surface to the last carbon of the alkyl chain (the methyl group).

At lower coverages, nearly all molecules are lying down with peak maximum between 75° and 80°. As the coverage increases from 0.77 molecules nm^{-2} to 1.10 molecules nm^{-2}, an increasing fraction of molecules start to have tilt angles around 50°, because some molecules cannot find space to lie along the surface. This behavior is intensified for medium coverages, where we see a transition from most of the chains having tilt angles between 45° to 50°. At 2.4 molecules nm^{-2} coverage a significant share of molecules tends to be perpendicular to the surface, with tilt angles of less than 30°. For higher coverages, the tilt angles start moving to lower values below 20°. This trend is in accordance with earlier reports of tilt angle for other silane chains with different alkyl chain lengths[7].

We measure the length of the chain as the distance between the silane oxygen bonded to surface and last carbon of the alkyl chain (in an all-trans configuration), obtaining a value of 0.975 nm. We multiplied this value by the cosine of the mean angle for each coverage density (Fig. 6), obtaining an estimate for the mean molecule height in our simulated systems, as shown in the main text Fig. 2k. Comparing the latter to the ellipsometric thickness presented in the main text Fig. 2b we see that the mean molecule height overlaps the experimental thickness data from ellipsometry.

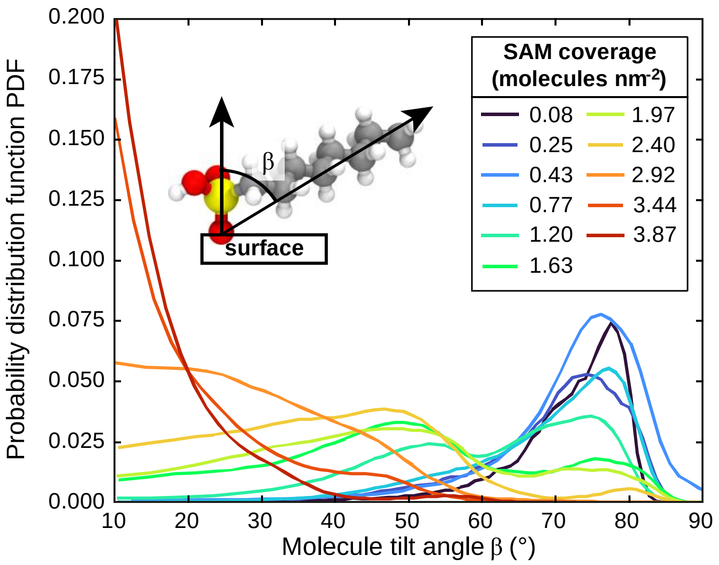

Fig. 6 | Angular distributions for all coverage densities simulated with MD. The inset shows how the tilt angle was calculated.

Supplementary Video 1 (Supplementary_Video_1.mov) | Thermal motion of OTS SAM at room temperature. The video represents SAM surface with 0.77 molecules nm^{-2} coverage over a time period of 10 ns.

Supplementary Note 8: Labelling OH vacancies with metal reactants

Let us first consider how the metal reactant compounds bind to the SAM surfaces during the vapor phase deposition step under the dry vacuum condition. The metal reactant compounds react with a surface OH group via reaction $\mathrm{ML_X + OH^* \rightarrow ML_{X-1}O^* + LH}$, where M is the central metal atom, L is ligand, X is the coordination number and $*$ marks surface group[8–11]. For diethylzinc (DEZ) $\mathrm{X} = 2$ and the reaction can occur for one ligand or for both of the ligands, if there are two adjacent OH-groups where a DEZ molecule can bind to (Fig. 7a). For titanium tetrachloride ($TiCl_4$), tetrakis(dimethylamido)hafnium (TDMAHf), and titanium tetraisopropoxide (TTIP) $\mathrm{X} = 4$ and the reaction can occur maximum three times due to molecule tetrahedral geometry, as one of the ligands is always pointing out of the surface (Fig. 7b). Once the surfaces are removed from the dry vacuum conditions, the remaining ligand(s) of the molecules react with moisture of the air $\mathrm{ML_{X-Y}O_Y^* + (X-Y)H_2O \rightarrow M(OH)_{X-Y}\,O_Y^* + (X-Y)LH}$ where Y is the number of remaining ligands. Therefore, the deposition of the metal reactant compounds effectively adds only metal atoms and OH groups to the SAM surface. Lastly, the density of OH groups on the surface is high, almost 10 groups nm^{-2} based on analysis done in the main text section “Quantification of OH vacancies of SAM surfaces”. Therefore, practically each vacancy of the SAM large enough for metal reactant adsorption contains at least one OH group allowing covalent bonding of the reactant to that vacancy.

The possible physisorption of metal reactants to the SAM surfaces needs to be considered, too. Based on earlier observations made by Hong *et al.*[12], adsorption of TDMAHf does not occur on top of a dense octadecyltrichlorosilane (ODTS) layer. OTS is chemically similar to ODTS, so it is safe to assume that TDMAHf does not physisorb on OTS. Adsorption of other metal reactants as function of OTS coverage resemble that of TDMAHf adsorption, implying that those reactants would not have physisorption either on top of the alkyl tails.

Physisorption of metal reactants could in principle occur also to the vacancies of the SAM. Possibility for this was checked by depositing DEZ on top of uncoated SiO_2 surface similarly as described in the Methods section. After deposition, the Zn/Si elemental ratio was determined with XPS at different temperatures starting from 20 °C and ending to 300 °C. Results are shown in Fig. 8, and no substantial desorption of Zn from the surface can be observed. The good temperature stability of the Zn/Si ratio indicates that DEZ is mainly covalently bonded to the surface. We note that the metal reactant deposition was also itself performed at 150 °C to minimize the possibility for physisorption of the reactant molecules. Lastly, the trend of metal/Si ratio is very similar for all metal reactants as function of SAM coverage, as is visible in the main text Fig. 3c. Had there been substantial physisorption of these reactants, it would be likely visible in the original XPS results, as all of the reactants have different ligands and thus likely different physisorption properties.

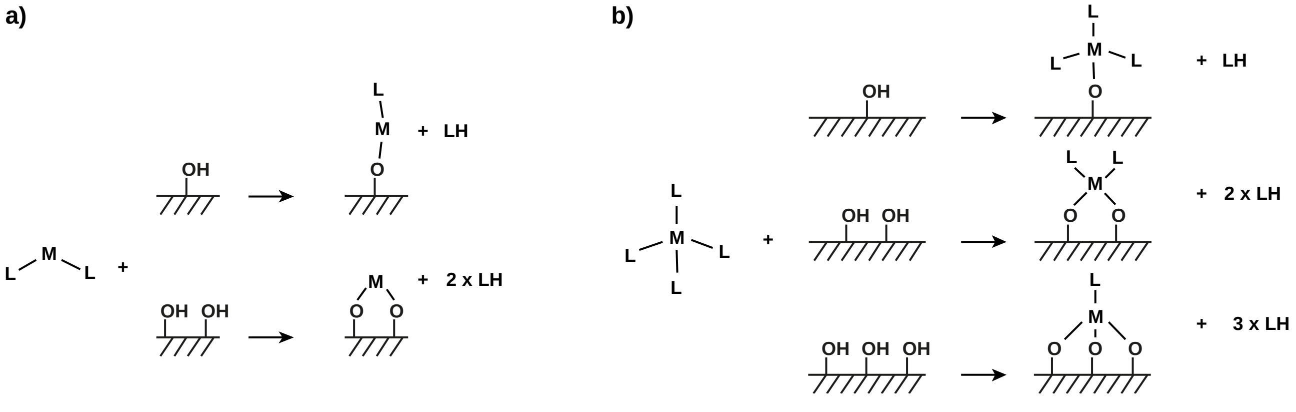

Fig. 7 | Metal reactants bonding to surface OH groups. Metal reactant **a)** with two ligands can bond to one or two adjacent surface OH groups and **b)** with four ligands can bind to one, two, or three adjacent OH groups. Molecule

geometry always causes one ligand to point away from the surface, thus preventing it from bonding to surface OH groups.

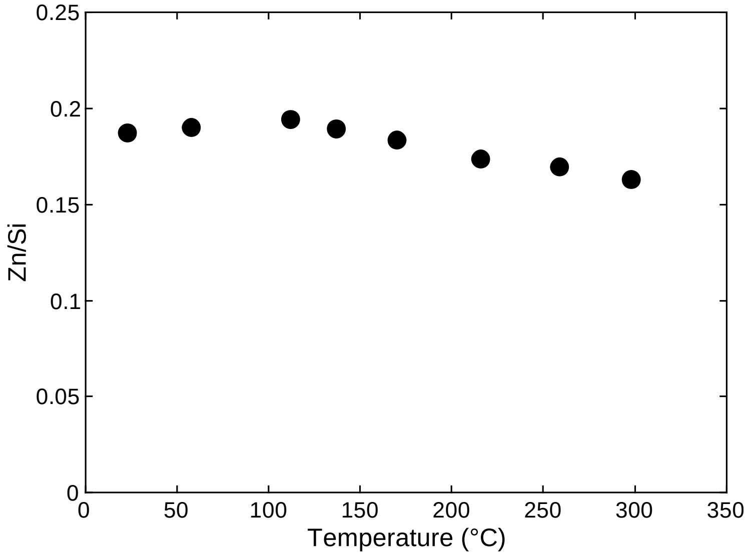

Fig. 8 | Elemental ratio of Zn to Si resolved by XPS as function of substrate temperature. The error margins related to Zn/Si ratio are difficult to quantify (as they originate from multiple sources and are related to temperature measurement location, data fit accuracy etc.) and are thus not drawn in the figure.

Supplementary Note 9: Details of XPS analysis

High-resolution x-ray photoelectron spectroscopy (XPS) was used to quantify the relative elemental concentrations of the SAM surfaces with labelling molecules. Each surface has four different elements: carbon (C), oxygen (O), silicon (Si) and the metal of the labelling molecule (Zn, Ti or Hf), see Fig. 9. Any other elements were below the reliable detection limit of XPS device. The amount of carbon is high even for samples without SAM layer. This is accounted to be due to airborne carbon contamination. Therefore, the amount of metal adsorption is quantified using ratio metal/Si, as it is more independent on variation of carbon concentration than the determined elemental concentrations.

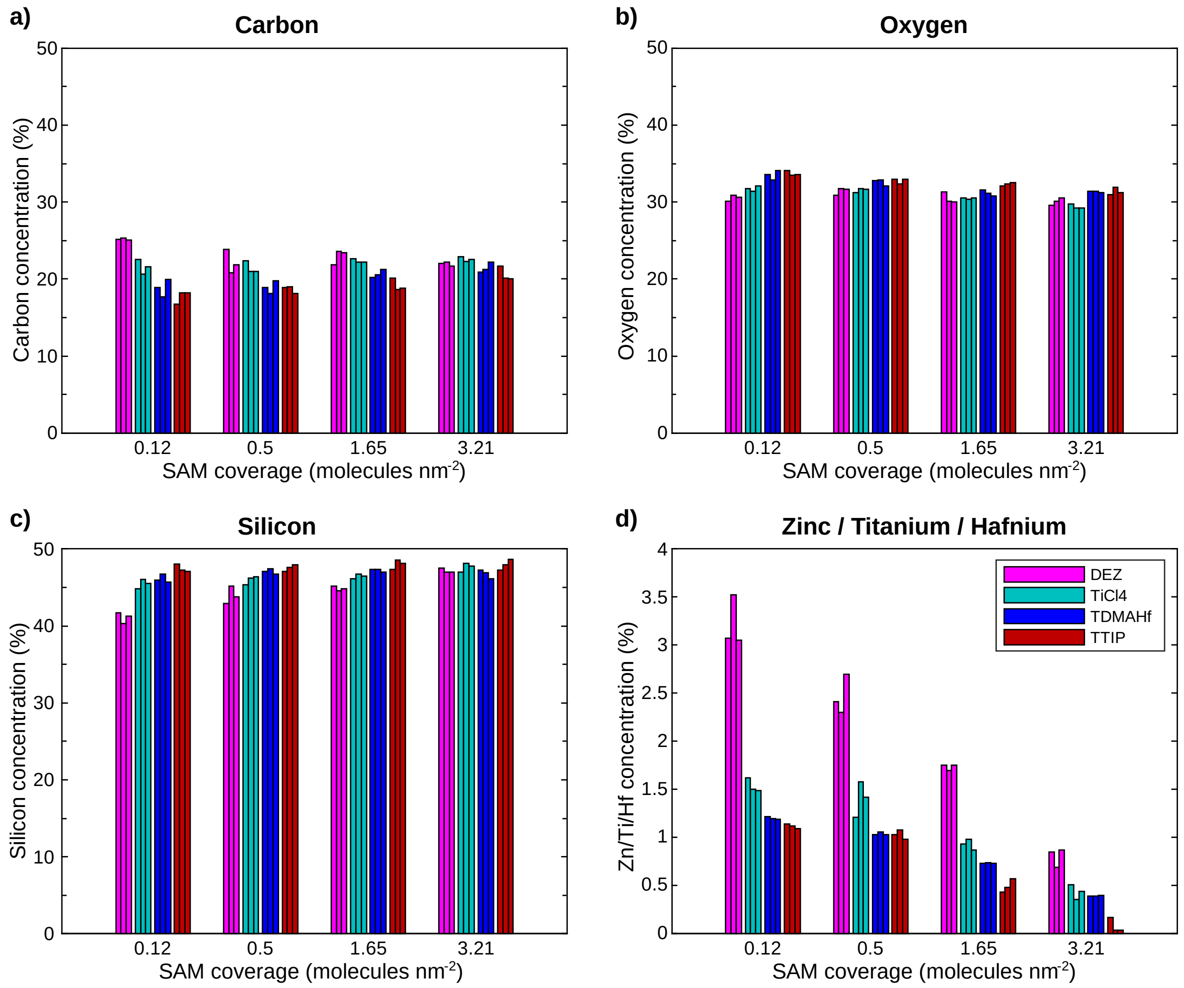

Fig. 9 | Elemental concentration of **a)** carbon (C), **b)** oxygen (O), **c)** silicon (Si), **d)** metal label (Zn/Ti/Hf) deposited on the SAM surface with varying SAM coverage. Elemental concentrations are measured from three different locations from each sample surface, which is represented by the three separate bars in each group.

Supplementary Note 10: Estimation of accessible OH groups from RBS analysis

Fig. 10 shows areal density of adsorbed TDMAHf labels on the SAM surfaces detected with Rutherford back scattering (RBS). According to RBS, there are 3.1 TDMAHf labels nm^{-2} on plain Si, corresponding to 0.32 nm^2 per a TDMAHf label. This average area per label molecule is much less than the estimated molecular cross-sectional area of a TDMAHf with all its ligands (0.82 nm^2). To fit a TDMAHf molecule to 0.32 nm^2 area, it needs to lose three of its dimethylamido ligands, when molecular cross-sectional area drops below 0.32 nm^2. Therefore, plain SiO_2 would have 9.3 OH groups nm^{-2}, if all adsorbed TDMAHf labels have bonded to three OH groups each and no major share of OH groups were left unlabeled. 9.3 OH groups nm^{-2} is also in line with the fact that plain Si can facilitate at least 3.9 OTS nm^{-2}, as OTS can bond up to three surface OH groups, which would require maximum amount of 11.7 OH groups nm^{-2} in case that all OTS molecules would bond to three OH groups each.

As SAM is applied on the Si, density of accessible OH groups decrease due to adsorption of OTS molecules. The adsorption of SAM also decreases the average number of adjacent free OH groups on the surface, and less TDMAHf molecules can bond to three OH groups each. With high SAM coverage, it becomes more likely that TDMAHf molecules bond to single OH group each. The experimental proof of this hypothesis is beyond the scope of this article, so we focus on estimating the possible range of accessible OH groups that limits in between the min and max shown in the main text Fig. 3d based on the adsorption of TDMAHf label molecules.

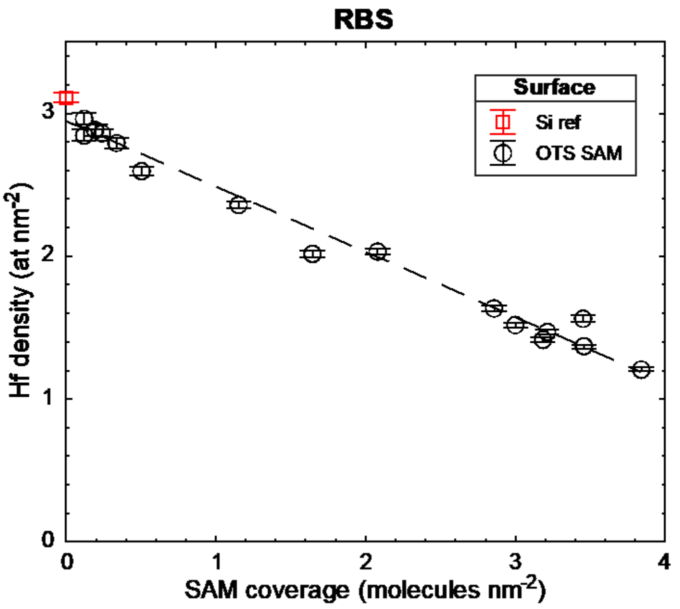

Fig. 10 | Areal density (coverage) of TDMAHf labels detected with RBS. The red data point represents TDMAHf adsorption on plain SiO_2 without SAM. The dashed line represents linear fit to the data. Each data point represents single measurement of the sample and error bars represent signal uncertainty as explained in Methods in the main text.

Supplementary Note 11: MD simulations of droplets on SAM surfaces

Droplet generation

We further used our MD-simulated SAM surfaces (Supplementary Note 18) for wetting simulations. In this case, to accommodate a water droplet in the simulation box, we replicated the surfaces of Supplementary Note 18 in the *x* direction 8 times (16 times in the 0.08 molecules nm^{-2} case), forming a surface of dimensions 26.7 nm x 3.5 nm. We placed 3493 water molecules as a droplet at about 1 nm above the surface, as shown in Fig. 11. Here we followed a common approach used in large-scale wetting simulations with MD: the system is periodic in all directions, but in the *y* direction, the water droplet extends from edge to edge of the simulation box. This makes the droplet assume an infinite semi-cylindrical shape in contact with the surface, also reducing errors due to line tension[13–16]. The initial system consists of a pre-formed semi-cylindrical shaped droplet to decrease the amount of equilibration time. We ran each SAM coverage system for 20 ns in the NVT ensemble, at 300 K and timestep of 1 fs. The first 15 ns were discarded from the analysis, which was enough time for the droplets to equilibrate on each of the surfaces and get to a stable configuration. We also ran the corresponding simulations on a plain SiO_2 surface for 40 ns.

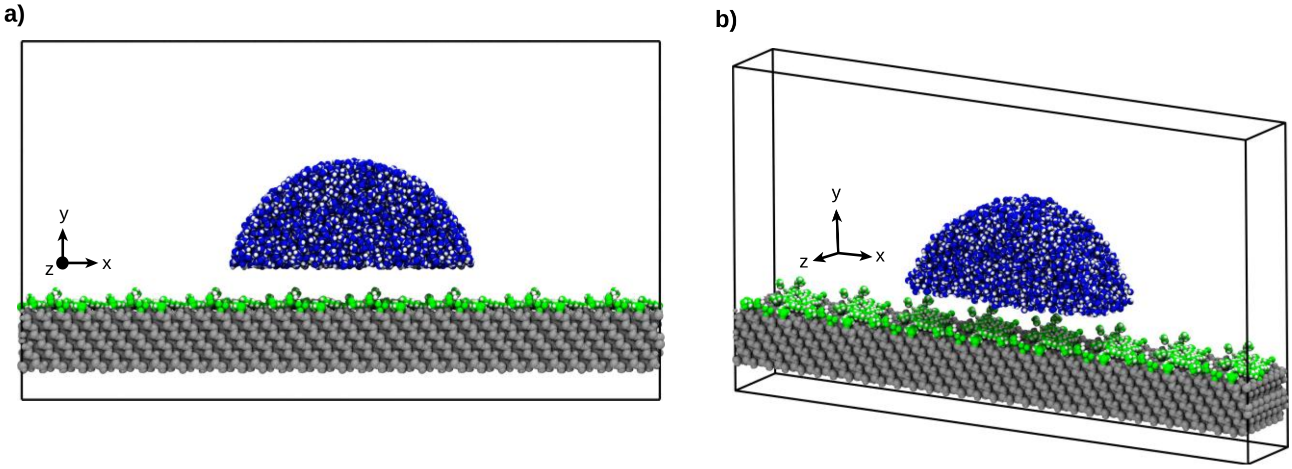

Fig. 11 | Initial configuration for the SAM coverage density of 0.77 molecules nm^{-2}. a) The *xy*-plane cross-section view and **b)** perspective view showcasing the semi-cylindrical shape of the droplet. Other SAM coverage densities follow the same configuration. Carbon, silicon, and oxygen atom of OTS molecules are in green. OTS hydrogens are in white. The SiO_2 substrate (silicon, oxygen, and hydrogen atoms) are grey. Oxygen atoms of water molecules are in blue. Hydrogen atoms of water molecules are in white.

Number and mass density of simulated droplets

Density maps of equilibrated droplets were calculated with the MDAnalysis python library, by dividing the simulation box in voxels of 1x1x1 Å^3 and then calculating the number density of oxygen atoms from water molecules in each of them. We used the last 500 frames (5 ns) of the simulations for coverages up to 2.92 molecules nm^{-2}. For coverages of 3.44 molecules nm^{-2} and 3.87 molecules nm^{-2} we used the last 50 frames, as in these simulations the droplets are highly mobile, and it was not possible to obtain well-defined density maps for longer times. Number density (Å^{-3}) was converted then to mass density (g cm^{-3}). Fig. 12a-l shows the mass density maps for the plain SiO_2 and each of the SAM coverage densities studied.

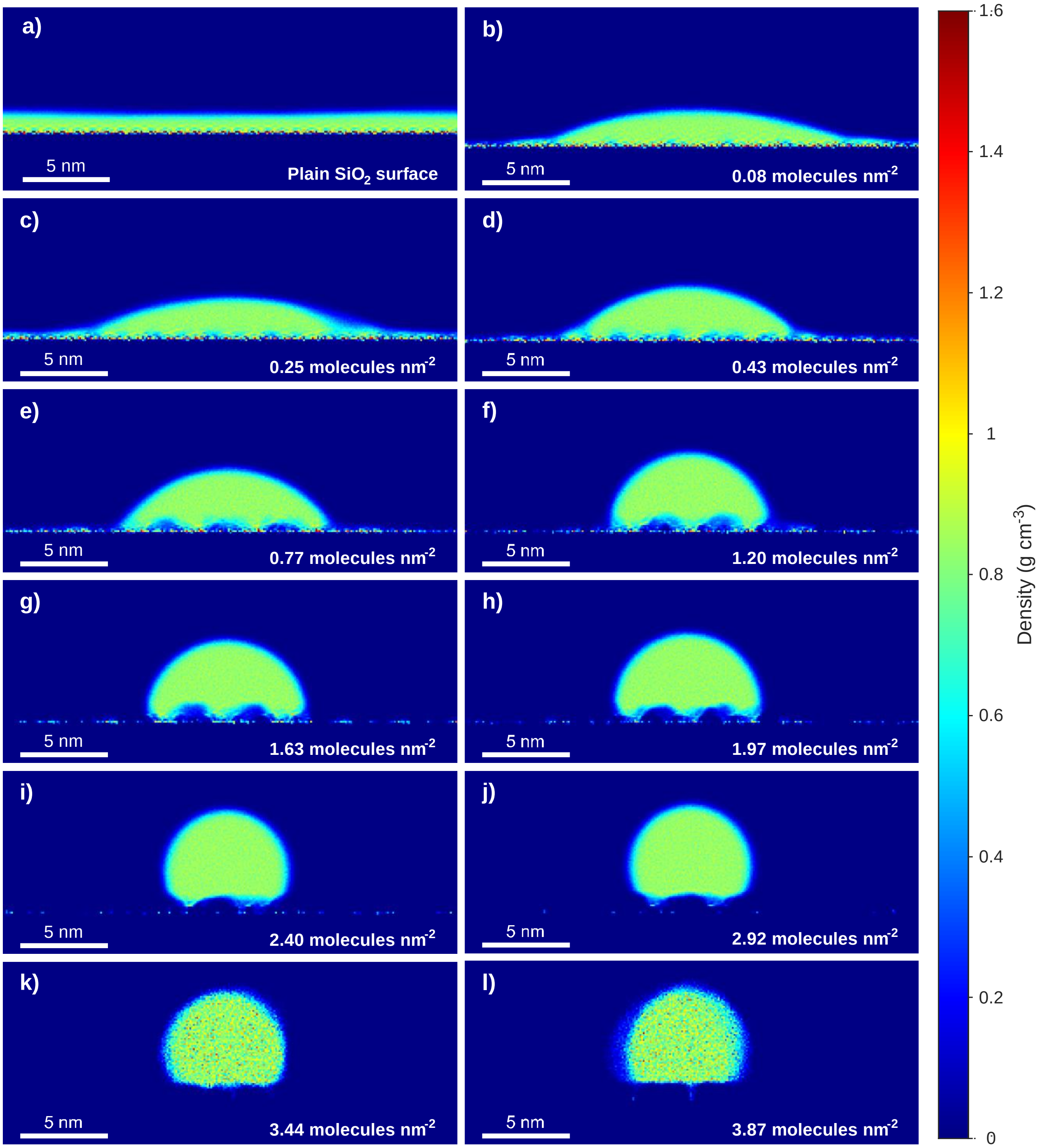

Fig. 12 | Mass density maps for the oxygen of water droplets on SAM surfaces. a) Water on plain SiO_2 as reference. **b-l)** Water droplet on SAM surfaces with coverages 0.08 – 3.87 molecules nm^{-2}. The color bar represents mass density in g cm^{-3} of the oxygen from the water molecules. For SAM up to coverage of 2.92 molecules nm^{-2}, the densities calculated averaging the oxygen atom positions of the last 5 ns of the simulations. For SAM coverages of 3.44 molecules nm^{-2} and 3.87 molecules nm^{-2}, the average was done in the last 0.25 ns of the simulations due to the high mobility of the droplets in those cases. For plain SiO_2, the average was done in the last 15 ns of the simulations.

Determination of contact angles

We determined the droplet's contact angle for each MD system. Due to the droplet's lateral movement in some of the systems, we reprocessed the trajectories to center the droplet on the same position in all frames and calculated the number density of water molecules for each configuration. The static contact angles of simulated droplets were obtained by fitting a circular arc into the edge of the droplet density profile and setting the baseline to the SAM average height, see Fig. 13a. Results are shown in Fig. 13b. The static contact angles agree with experimentally obtained ACA for coverages below 2.0 molecules nm^{-2}. Above that coverage, simulated droplets have higher static contact angle than the experimentally obtained ACA. This is due to the relatively high surface roughness in comparison to droplet size in the MD simulations. Contact angle dependency on the simulated droplet size was checked with droplets ranging in size from 1718 to 4274 water molecules without notable size sensitivity observed (see Fig. 13c).

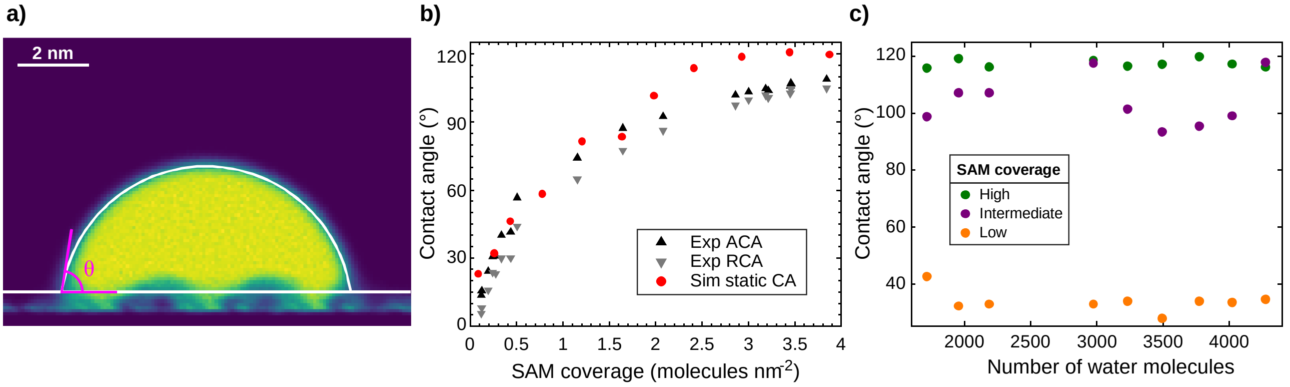

Fig. 13 | Comparison of experimental ACA and RCA to static CA of simulated droplet on OTS SAM surfaces. a) Schematics showing how CA were determined from simulated droplets. The white lines represent the circular arc fitted to the droplet number density profile and the SAM average height above the SiO_2 surface. **b)** Experimentally determined advancing and receding contact angles compared to contact angles calculated based on the MD simulations. **c)** Contact angles obtained from molecular dynamics simulations for varying water droplet sizes. Errors of the data points vary between 1.4° and 7.9° depending on system, yet not presented due to lack of statistics making error estimation imprecise.

Supplementary Note 12: Prediction of SAM contact angle with Cassie's law

The contact angle of a chemically heterogenous, smooth surface can be predicted with Cassie's law

$$\cos\theta_{\mathrm{Cassie}} = \sum_i f_i \cos\theta_i \tag{5}$$

where f_i is the fraction of surface component i and θ_i is its contact angle. For a case where SAM is grown on well cleaned, hydroxyl rich SiO_2 surface, Cassie's law can be written as

$$\cos\theta_{\mathrm{Cassie}} = f_{\mathrm{SAM}}\cos\theta_{\mathrm{OTS}} + (1 - f_{\mathrm{SAM}})\cos\theta_{\mathrm{SiO2}} \tag{6}$$

where f_{SAM} represents the normalized surface coverage of SAM, θ_{OTS}contact angle of homogenous OTS SAM surface and θ_{SiO2} contact angle of homogeneous SiO_2 surface. Here we used θ_{OTS} = 110° and θ_{SiO2} = 0°. For surface coverage f_{SAM}, two different estimators were tested. The first one is the SAM coverage obtained from ellipsometry normalized by its maximum value. The second one is the covered area of SAM alkyl chains (see Fig. 14a) that is calculated from areal molecule density and average alkyl tilt angle

$$A_{\mathrm{SAM}} = \left(\pi R_{\mathrm{alkyl}}^2 \cos\beta + 2R_{\mathrm{alkyl}} H_{\mathrm{alkyl}} \sin\beta\right) f_{\mathrm{SAM}} \tag{7}$$

where R_{alkyl} and H_{alkyl} are the radius and height of the SAM octyl tail, respectively, and β is the average molecule tilt angle as depicted in Fig. 6. R_{alkyl} = 0.25 nm and H_{alkyl} = 1.0 nm were used in the calculations. As tilted molecules may partially lie on top of each other, the covered area can be larger than the underlying area itself, and to be used as a coverage estimation in Cassie's law it requires normalization with its maximum value. Fig. 14b shows the difference in f_{SAM} calculated by the two methods.

Fig. 14c shows the measured ACA and RCA of the SAM surfaces and the contact angle for each surface predicted with Cassie's law using the two above mentioned coverage estimators. As is seen from the graph, the areal density of SAM alone fails to predict the OTS SAM contact angle, especially with coverage of 0.5 molecules nm^{-2} and above. Considering the average molecule tilt, the prediction becomes better. The high tilt of molecules at low coverages increases the surface contact angle at early stage of deposition and lowering of the tilt in the later stage of growth explains the slower growth of contact angles. A note must be made that the simple normalization of the covered area is far too naive method as it ignores the varying fraction of molecules lying on top of each other on different SAM coverages, and thus Cassie angles calculated using this coverage estimator cannot be expected to be fully accurate.

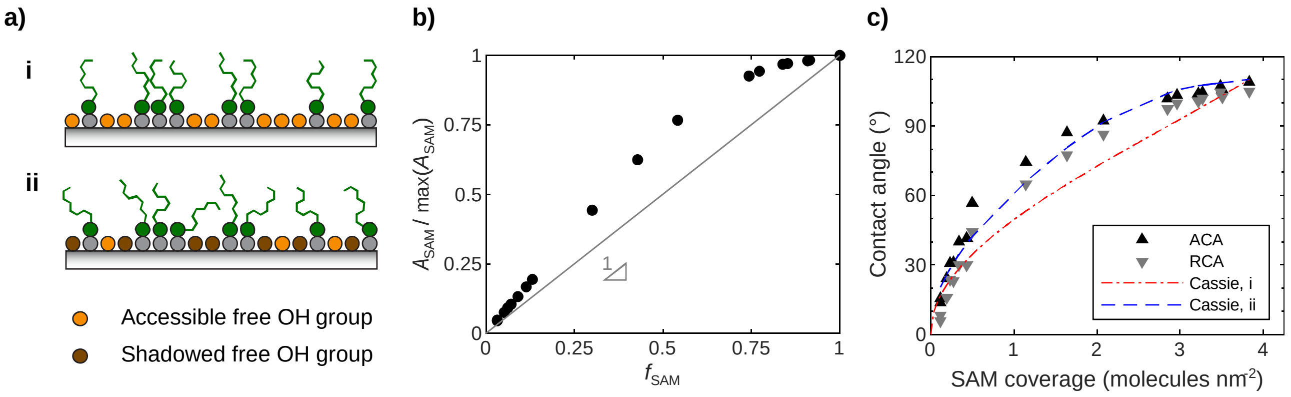

Fig. 14 | Prediction of OTS SAM surface contact angle. a) Illustration of accessible OH groups i) without molecule tilt and ii) with average molecule tilt considered. **b)** Normalized covered area of SAM molecules as function of normalized SAM coverage. The solid grey line represents 1:1 ratio. **c)** Experimentally observed contact angles and Cassie's law prediction for the contact angles with the two coverage estimators.

Supplementary Note 13: Sliding angle measurements

Conversion from sliding angles to contact line friction

Droplet sliding angles were used for characterizing the contact line friction of the SAM surfaces. The results are shown in Extended Data Figure 1b. Based on balance of forces, droplet starts sliding when droplet gravity component parallel to the surface becomes larger than the contact line friction force F_{μ}. Therefore, at the minimum sliding angle α applies

$$F_{\mu} = V\rho g \sin\alpha \quad (8)$$

where V is droplet volume, ρ water density (1.0 g cm^{-3} used here), and g is the constant of gravitational acceleration (9.81 m s^{-2} used here). The contact line friction force obtained from the sliding angle measurements is shown in the Extended Data Figure 1d. The droplet volume used in the calculation was read from its sideview profile and by assuming cylindrical symmetry. As the contact line friction force is dependent on the droplet size, the obtained friction was further normalized with the droplet contact region diameter D, i.e., calculating F_{μ}/D values. Extended Data Figure 1e shows the results and that the data collected with three different droplet volumes have approximately equal F_{μ}/D values as function of SAM coverage.

Supplementary Video 2 (Supplementary_Video_2.mov). Sliding angle experiment of 10 μl droplet on OTS SAM with 0.12 molecules nm^{-2} coverage. The camera tilts along the sample, so sample surface does not tilt visibly. The playback speed is 5x real time.

Supplementary Video 3 (Supplementary_Video_3.mov). Sliding angle experiment of 10 μl droplet on OTS SAM with 0.50 molecules nm^{-2} coverage. The camera tilts along the sample, so sample surface does not tilt visibly. The playback speed is 5x real time.

Supplementary Video 4 (Supplementary_Video_4.mov). Sliding angle experiment of 10 μl droplet on OTS SAM with 3.2 molecules nm^{-2} coverage. The camera tilts along the sample, so sample surface does not tilt visibly. The playback speed is 5x real time.

Droplet sliding with constant tilt angle

Main text Fig. 4b shows how droplets slide as function of surface tilt angle. Fig. 15 shows how droplets slide with a constant tilt angle that is slightly above the minimum sliding angle for droplets. The sliding is slow as the driving force for the sliding (the droplet gravity component parallel to the surface) is only slightly larger than the contact line friction and the remaining droplet acceleration is likely consumed by viscous losses. Due to the slow movement the droplets also have time to evaporate, which reduces the driving force for the sliding and thus the droplet movement slows down over time.

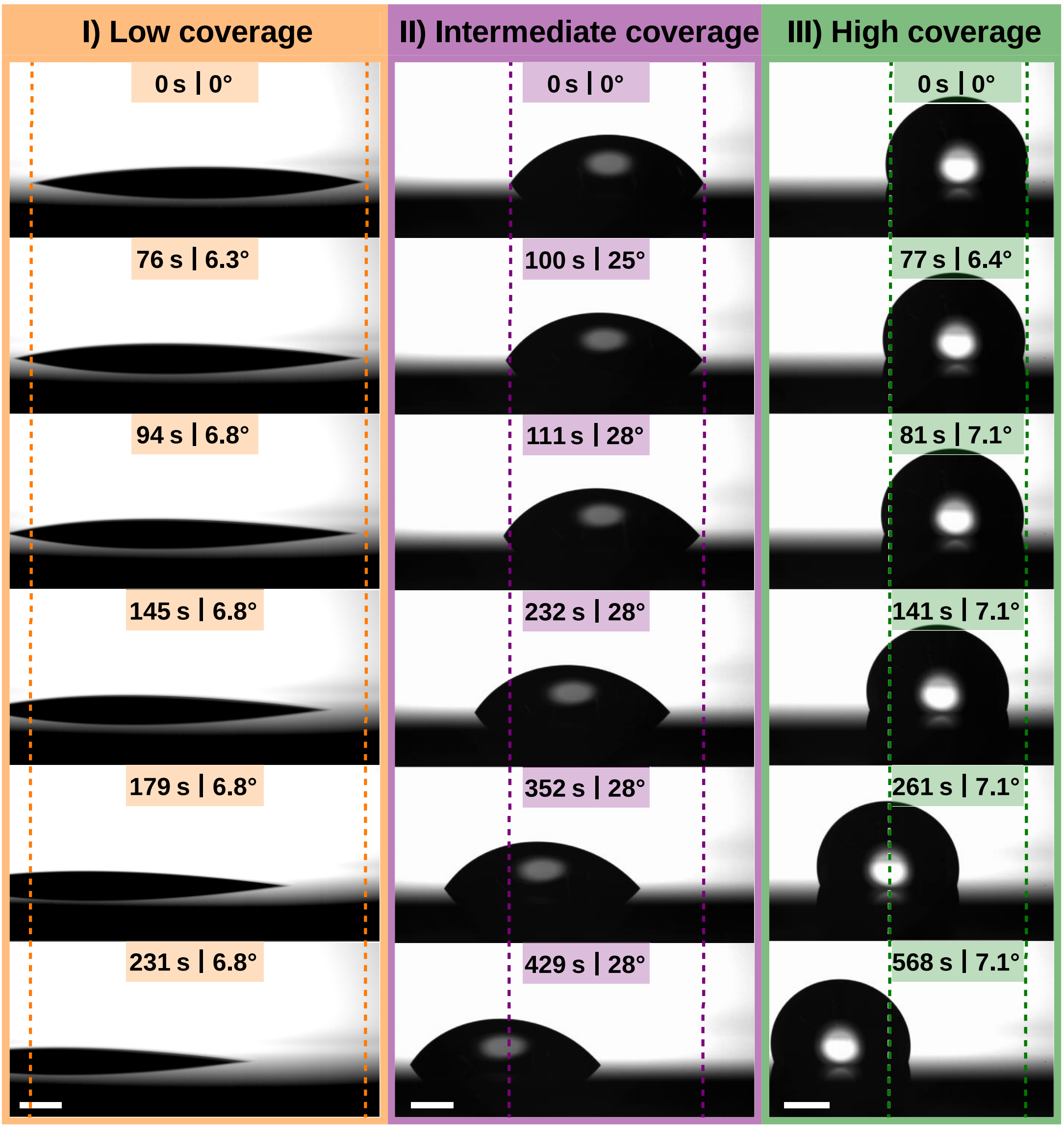

Fig. 15 | Droplet sliding with constant surface tilt. The surfaces are first tilted until droplet starts sliding, after which the tilting is halted and droplets let to slide on the surfaces.

Supplementary Note 14: Mobility of interfacial water molecules via residence time analysis

We calculated the density profile of water-oxygen for each of the equilibrated systems (i.e., without any external force in x-direction), as shown in Fig. 16. For coverages up to 2.92 molecules nm^{-2}, we can see that there is a large first hydration layer (HL), which is the first peak starting from substrate. This peak is due to the water molecules interacting with the OH groups on SiO_2. We note that this happens both at the droplet's contact area as well as around the droplet on the surface — Fig. 12 shows in the low-coverage regime a thin water film extending from the droplet's contact line along the SiO_2. As the SAM coverage increases, the first HL progressively diminishes, as more OH groups are substituted for OTS molecules. At 1.97 - 2.92 molecules nm^{-2}, we can see that the first HL is less pronounced, and although the SAM already forms a surface (indicated by the increased water-oxygen density around 1.5 nm) there is no HL formed at the SAM-water interface. At the higher coverages however, we can see that the first HL at the silica surface vanishes completely, and the first HL is on top of the SAM.

The first hydration layer can give us important information about the water dynamics at the droplet's contact line. To access this, we calculated the residence time of water molecules in the first HL for each of the systems (Fig. 17). The residence correlation function[17] calculates how long an atom stays in a determined region of the simulation box via the time correlation function in Equation 9:

$$P(\tau) = \frac{1}{T}\sum_{t=1}^{T}\frac{N(t,\, t+\tau)}{N(t)} \qquad (9)$$

where T is the maximum time of the simulation, τ is the timestep, $N(t)$ is the number of atoms at time t, and $N(t,\, t+\tau)$ is the number of atoms at every simulation frame from t to τ. The correlation decay allows determining the residence time of the atoms in a determined region: the faster the decay the shorter the average residence time is.

For analysis, we tracked oxygen atoms of water molecules at the first HL in each of the SAM surfaces. We considered the same thickness (0.3 nm) in all cases. Up to 2.92 molecules nm^{-2} (and for plain SiO_2 as well) we considered the first HL as being at the water-SiO_2 interface. For SAM coverages of 3.44 molecules nm^{-2} and 3.87 molecules nm^{-2} we considered the thickness of 0.3 nm at the water-SAM interface, which is around 1.5 nm in the plot of Fig. 16. For these latter two concentrations there is almost no water closer to the silica and the residence time analysis is not able to be performed at the SiO_2.

The residence time indicates how fast is the exchange of water molecules at the first HL in each of the coverages. Fig. 17 shows that the residence time decays fastest for coverage range 3.44 – 3.87 molecules nm^{-2} meaning that water molecule exchange is fastest on the SAM layer. For SAM coverage from plain SiO_2 to 2.92 molecules nm^{-2} the water molecule exchange rate depends on the available SiO_2 area. The higher the SAM coverage, the more barriers there are restricting water molecule movement, which slows down water molecule exchange rate. For plain SiO_2, there are no alkyl chains as barriers and water molecule exchange rate is highest.

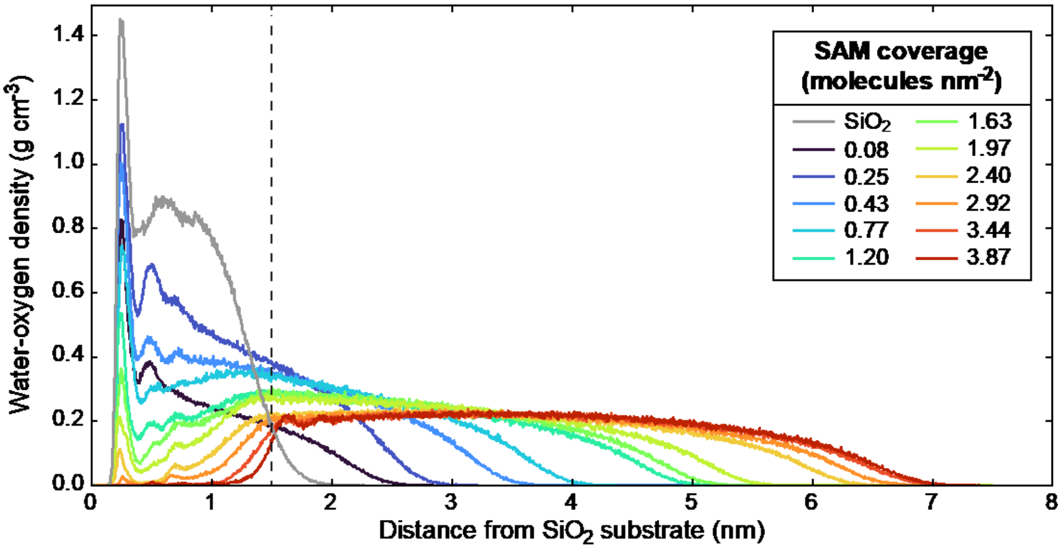

Fig. 16 | Mass density profiles of water molecule oxygen atoms along the SAM-SiO_2 surface normal. The silica substrate edge is located at 0 nm, and it is defined by the z-coordinate position of topmost Si atom layer of the substrate. The vertical dashed black line indicates the location of the SAM edge for the coverage of 3.87 molecules nm^{-2}, which is the maximum coverage studied in this work.

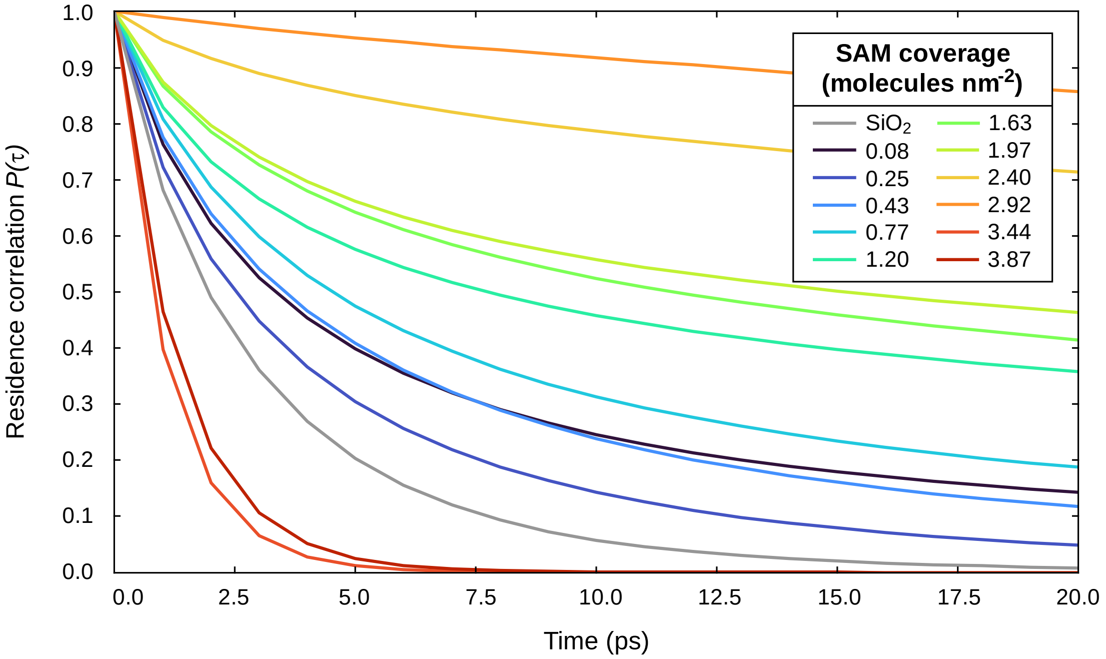

Fig. 17 | Residence correlation *P(τ)* of water molecule oxygen atoms in the first hydration layer for each of the SAM coverages. The correlation decay allows determining the residence time. Plain SiO_2 and SAM coverages 0.08 – 2.92 molecules nm^{-2} were analyzed at the SiO_2 interface and SAM coverages 3.44 – 3.87 molecules nm^{-2} were analyzed above the SAM interface.

Supplementary Note 15: MD simulation of droplet sliding

We performed further MD simulations addressing droplet sliding by applying a force of 0.001 kcal/mol/Å (6.95×10^{-5} μN) in the *x* -direction. The force was applied on each oxygen atom of the water molecules in the droplet after equilibration in each of the systems. These simulations were run for 5 ns in the NVT ensemble at 300 K. A timestep of 1 fs was used. The simulations give important insights into the mechanism of contact line friction. However, we must note that droplet speed does not directly correspond to contact line friction due to scale difference, diffusion considerations within the employed simulation model, and the relatively high speed of the droplets (in the order of 10 – 100 m s^{-1}) in the MD simulations. Movies 5-7 show how water droplets move on low-, intermediate- and high-coverage SAMs, respectively.

Supplementary Video 5 (Supplementary_Video_5.mov) | MD simulation of droplet sliding on OTS SAM with 0.25 molecules nm^{-2} coverage. The video shows a droplet with 3493 water molecules moving on the OTS SAM surface over a time period of 5.0 ns.

Supplementary Video 6 (Supplementary_Video_6.mov) | MD simulation of droplet sliding on OTS SAM with 0.77 molecules nm^{-2} coverage. The video shows a droplet with 3493 water molecules moving on the OTS SAM surface over a time period of 5.0 ns.

Supplementary Video 7 (Supplementary_Video_7.mov) | MD simulation of droplet sliding on OTS SAM with 3.44 molecules nm^{-2} coverage. The video shows a droplet with 3493 water molecules moving on the OTS SAM surface over a time period of 1.2 ns.

Supplementary Note 16. Comparison of contact line friction of SAM on smooth and bSi surfaces

There are two major differences in wetting properties of OTS SAM when grown on flat Al_2O_3 and SiO_2 surfaces. Firstly, ACA and RCA develop much faster from 0° (freshly ALD grown Al_2O_3 is superhydrophilic) to above 90°, see Fig. 18. Secondly, CAH is much higher for partially complete SAM grown on Al_2O_3 than on SiO_2. As a result, SAM grown on Al_2O_3 has larger range of CAH such that ACA and RCA are both above 90° than SAM grown on SiO_2 as function of SAM growth time. Therefore, bSi surfaces with Al_2O_3 + SAM surface chemistry are superhydrophobic (on which droplets remain in Cassie-Baxter state due to high contact angles) with wide range of SAM growth times and have a wide range of SAM chemical heterogeneity (CAH of SAM). With SiO_2 + SAM surface chemistry, only long SAM growth times enable Cassie-Baxter state for droplet, but then the range of chemical heterogeneity is narrow. Therefore, we selected to compare contact line friction of SAM grown on flat Al_2O_3 and Al_2O_3 coated bSi, and to ensure that droplets are in the Cassie state on the Al_2O_3 coated bSi, we only use SAM growth times of 10 min and longer in the comparison.

Normalized CLF of SAM on the flat Al_2O_3 we measured via contact angle goniometry and via Equation 10

$$\frac{F_{\mu}}{D} = \frac{24}{\pi^3}\gamma(\cos\theta_{\mathrm{REC}} - \cos\theta_{\mathrm{ADV}}) \tag{10}$$

where D is droplet contact region diameter, γ is water surface tension (γ = 72.8 mN m^{-1} used here for water), and θ_{REC} and θ_{ADV} receding and advancing contact angles, respectively[18]. CLF of SAM on the Al_2O_3 coated bSi was measured with the micropipette force sensor (MFS). Friction was measured for each of the tested surfaces from multiple locations using multiple droplet sizes, and linear fit to the data was performed to obtain the normalized CLF (Fig. 19).

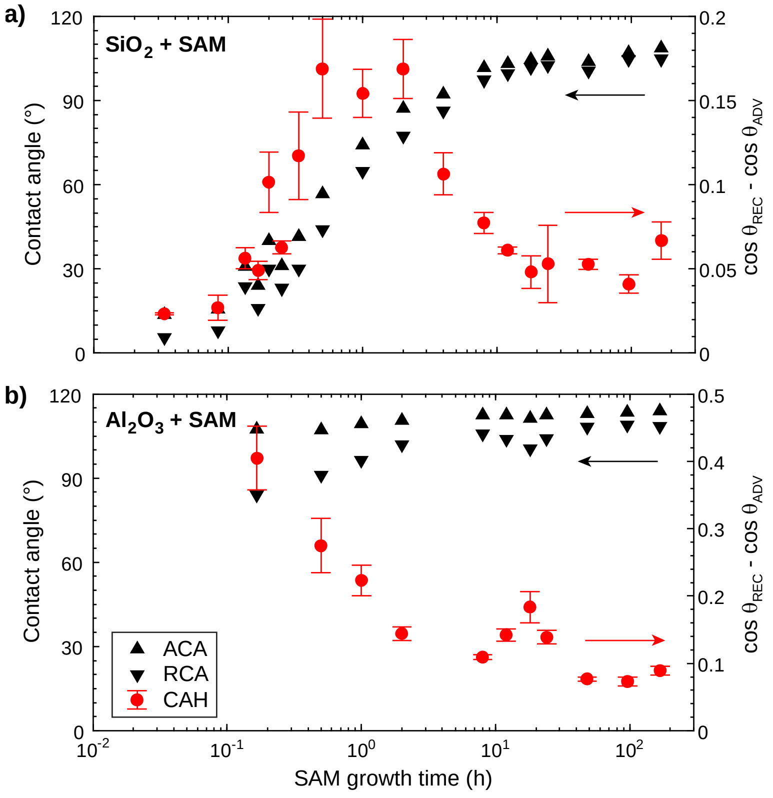

Fig. 18 |. Contact angles of SAM surfaces grown a) on SiO_2 and b) on Al_2O_3. Error bars represent standard deviation of average contact angle hysteresis recorded from three locations from the sample. Error bars for ACA and RCA are excluded due to their small scale.

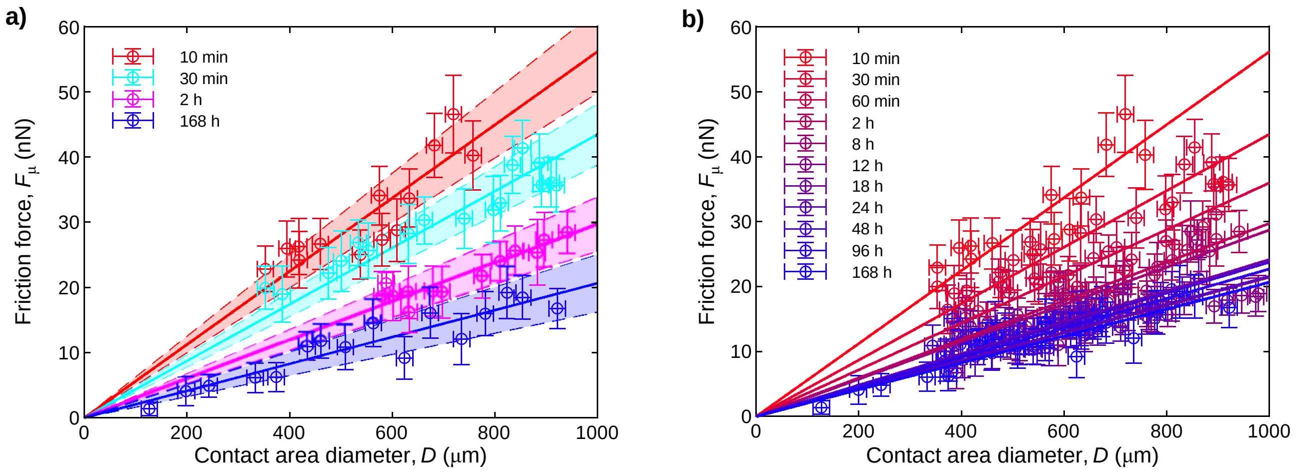

Fig. 19 | Friction force measurements of OTS SAM coated bSi surfaces. Average friction of **a)** four OTS SAM coated bSi surfaces and **b)** all surfaces as function of droplet size (contact area diameter). In both panels legend shows OTS SAM growth time. Each data point represents an average friction obtained from a single MFS scan as is presented in Fig 6c of the main text. Vertical error bars represent standard deviation of friction over the MFS scan averaged region, and horizontal error bars represent 16 μm accuracy limit of contact region diameter determination. Solid lines represent linear fits to data series, and their slope corresponds to normalized CLF F_μ/D. In a) shadowed areas represent 95% confidence intervals for linear fits (shadowed areas are left out from panel b due to figure clarity). Number of measurements n = 13, 17, 21, 13, 20, 24, 13, 19, 25, 16, 16 (n reported in ascending order in terms of SAM growth time) used for linear fits.

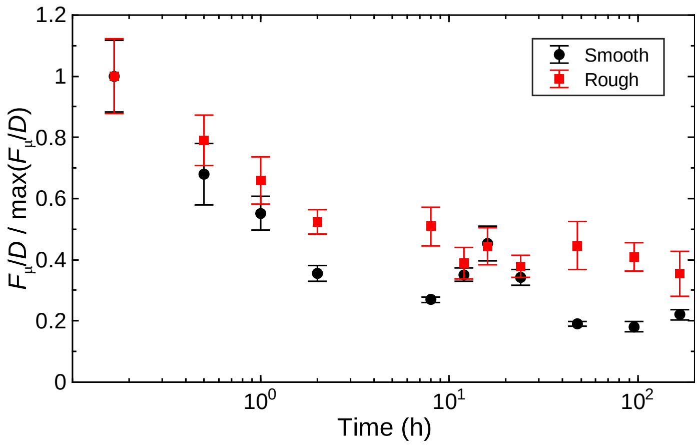

Fig. 20 | Relative change of droplet contact region normalized friction force of SAM on smooth and rough substrates. For both data series, the data point value is obtained by dividing the F_μ/D value with maximum F_μ/D value of that series. The error bars of smooth data series represent standard deviation of the measurement average (n = 5), and the error bars of the rough data series represent the 95% confidence interval of linear fit to F_μ vs. D data (n = 13, 17, 21, 13, 20, 24, 13, 19, 25, 16, 16, see Fig. 19).

Supplementary Video 8 (Supplementary Video 8.mov) | Example of micropipette force sensor (MFS) scan of SAM coated black silicon surface. The video is from the bSi surface with OTS SAM grown for 48 h. The droplet volume is ca. 10 μl and total scan distance approximately 2 mm. In the video droplet and camera are kept stationary while the surface is moved towards left.

Supplementary Note 17: Determination of advancing and receding contact angles

Advancing and receding contact angle measurements were performed adapting the protocol published by Huhtamäki *et al.*[19] as follows. First, about 10 µl droplet is deposited on the surface. Next, 1 µl is added to the droplet at slow rate of 0.05 µl s^{-1}. After this, video recording with a sideview camera is started, and droplet volume is increased further 9 µl at the slow 0.05 µl s^{-1} rate to record ACA, see Fig. 21. After that, 10 µl is first added to the droplet and then 8 µl is removed from it to set the droplet contact angles close to RCA. Then 1 µl is withdrawn from the droplet with slow rate of 0.05 µl s^{-1}. After this, 9 µl is withdrawn from the droplet at slow rate of 0.05 µl s^{-1} while recording the droplet sideview profile to capture RCA, see Fig. 21.

Young-Laplace fitting is used to obtain left and right contact angles and droplet baseline length for each recorded frame. Advancing contact angle is obtained by fitting a linear line to the mean contact angle (mean of left and right contact angles) data and calculating the fit value when the droplet contact line starts advancing (marked with i in Fig. 21, the moment when baseline length start increasing). The CA recording is kept on for longer time (3 min) to ensure that there were no defects near the droplet during the CA recording. RCA is obtained similarly from the moment when droplet contact line starts receding (marked with ii in Fig. 21).

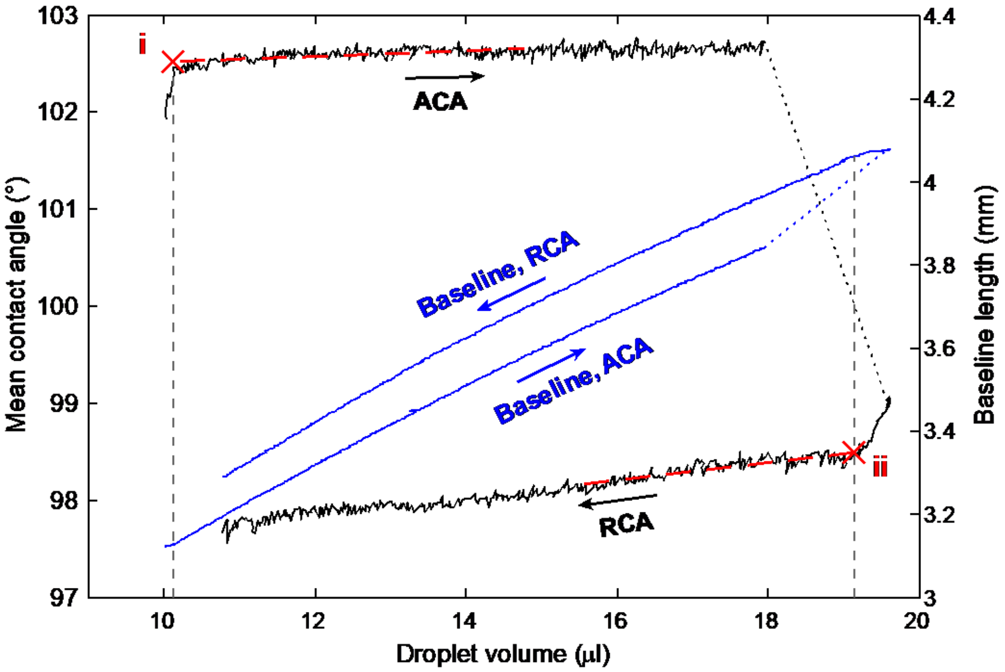

Fig. 21 | Procedure for determining advancing and receding contact angles. The CA values represent mean from left and right contact angles. Linear fits to the CA data are marked with red dashed lines and fitted values for ACA and RCA are marked with red crosses: i for ACA and ii for RCA.

Supplementary Note 18: Details of MD simulation of OTS SAM assembly on SiO_2

The simulation model consisted of an α-quartz surface, adapted from Emami *et al.*[20] This specific substrate was chosen because it gives good agreement with contact angles for silica substrates (contact angle of 0°), the same behavior found in our experiments. The surface is composed of one OH group per surface Si (concentration of 4.81 OH nm^{-2}).

We used a similar approach as described by Castillo *et al.*[7] and Roscioni *et al.*[21] in constructing the OTS SAM on silica. An OH was removed from the silica substrate and one H was removed from the hydroxylated OTS chain. Then the unpaired O from OTS was bound to the unpaired Si from the silica. The net charge of the simulation system was kept zero by neutralizing the resulting charge (generated by the removal of atoms) via corresponding opposite charge split between the three oxygens from the OTS chain.

To create the SAMs, we considered a silica slab of dimensions 3.3 nm x 3.5 nm with thickness of 2.4 nm. Different amounts of OTS molecules were then randomly placed on top of the silica substrate, with the same number of OH groups being removed from the silica at random sites. We first performed an energy minimization with the conjugate gradient method, followed by a NVT simulation at 400 K for 2 ns. During this stage, we used the "fix create/bonds" command from LAMMPS to create bonds on the fly between silane O and silica Si that had their H and OH missing, respectively. The chains quickly adhered to the surface at all the spots with missing OH groups. After that, we performed another run in the NVT ensemble cooling down the system from 400 K to 300 K during 0.5 ns.

It is important to notice that the intent of this protocol was to effectively generate the SAMs, not to study how their self-assembly process occurs, which would require longer simulations times as well as having other mechanisms and reactions. With this approach, we generated surface slabs of various coverage densities as shown in Table 3.

Table 3 | Number of OTS molecules simulated for dry conditions, with their respective coverage densities and OTS/OH ratios.

Number of OTS molecules	Coverage (molecules nm^{-2})	OTS/OH ratio (%)
**1**	0.08	1.78
**3**	0.25	5.35
**5**	0.43	8.92
**9**	0.77	16.07
**14**	1.20	25.0
**19**	1.63	33.92
**23**	1.97	41.07
**28**	2.40	50.0
**34**	2.92	60.71
**40**	3.44	71.42
**45**	3.87	80.35

Captions of Supplementary Videos

Supplementary Video 1 (Supplementary Video_1.mov) | Thermal motion of OTS SAM at room temperature. The video represents SAM surface with 0.77 molecules nm^{-2} coverage over a time period of 10 ns.

Supplementary Video 2 (Supplementary Video_2.mov) | Sliding angle experiment of 10 μl droplet on OTS SAM with 0.12 molecules nm^{-2} coverage. The camera tilts along the sample, so sample surface does not tilt visibly. The playback speed is 5x real time.

Supplementary Video 3 (Supplementary Video_3.mov) | Sliding angle experiment of 10 μl droplet on OTS SAM with 0.50 molecules nm^{-2} coverage. The camera tilts along the sample, so sample surface does not tilt visibly. The playback speed is 5x real time.

Supplementary Video 4 (Supplementary Video_4.mov) | Sliding angle experiment of 10 μl droplet on OTS SAM with 3.2 molecules nm^{-2} coverage. The camera tilts along the sample, so sample surface does not tilt visibly. The playback speed is 5x real time.

Supplementary Video 5 (Supplementary Video_5.mov) | MD simulation of droplet sliding on OTS SAM with 0.25 molecules nm^{-2} coverage. The video shows a droplet with 3493 water molecules moving on the OTS SAM surface over a time period of 5.0 ns.

Supplementary Video 6 (Supplementary Video_6.mov) | MD simulation of droplet sliding on OTS SAM with 0.77 molecules nm^{-2} coverage. The video shows a droplet with 3493 water molecules moving on the OTS SAM surface over a time period of 5.0 ns.

Supplementary Video 7 (Supplementary Video_7.mov) | MD simulation of droplet sliding on OTS SAM with 3.44 molecules nm^{-2} coverage. The video shows a droplet with 3493 water molecules moving on the OTS SAM surface over a time period of 1.2 ns.

Supplementary Video 8 (Supplementary Video_8.mov) | Example of micropipette force sensor (MFS) scan of SAM coated black silicon surface. The video is from the bSi surface with OTS SAM grown for 48 h. The droplet volume is ca. 10 μl and total scan distance approximately 2 mm. In the video droplet and camera are kept stationary while the surface is moved towards left.

Supplementary Information references

1. Fadeev, A. Y. & McCarthy, T. J. Self-Assembly Is Not the Only Reaction Possible between Alkyltrichlorosilanes and Surfaces: Monomolecular and Oligomeric Covalently Attached Layers of Dichloro- and Trichloroalkylsilanes on Silicon. *Langmuir* **16**, 7268–7274 (2000).

2. Sastry, M. A note on the use of ellipsometry for studying the kinetics of formation of self-assembled monolayers. *Bull. Mater. Sci.* **23**, 159–163 (2000).

3. Cuypers, P. A. *et al.* The adsorption of prothrombin to phosphatidylserine multilayers quantitated by ellipsometry. *J. Biol. Chem.* **258**, 2426–2431 (1983).

4. Schwartz, D. K. Mechanisms and kinetics of self-assembled monolayer formation. *Annu. Rev. Phys. Chem.* **52**, 107-137 (2001).

5. MacPhail, R. A., Strauss, H. L., Snyder, R. G. & Elliger, C. A. C-H Stretching Modes and the Structure of n-Alkyl Chains. 2. Long, All-Trans Chains. *J. Phys. Chem.* **88**, 334–341 (1984).

6. Snyder, R. G., Strauss, H. L. & Elliger, C. A. C-H Stretching Modes and the Structure of n-Alkyl Chains. 1. Long, Disordered Chains. *J. Phys. Chem.* **86**, 5145–5150 (1982).

7. Castillo, J. M., Klos, M., Jacobs, K., Horsch, M. & Hasse, H. Characterization of Alkylsilane Self-Assembled Monolayers by Molecular Simulation. *Langmuir* **31**, 2630–2638 (2015).

8. Cai, J. *et al.* A revisit to atomic layer deposition of zinc oxide using diethylzinc and water as precursors. *J. Mater. Sci.* **54**, 5236–5248 (2019).

9. Aarik, J., Aidla, A., Mändar, H. & Uustare, T. Atomic layer deposition of titanium dioxide from TiCl4 and H2O: investigation of growth mechanism. *Appl. Surf. Sci.* **172**, 148–158 (2001).

10. Hausmann, D. M., Kim, E., Becker, J. & Gordon, R. G. Atomic layer deposition of hafnium and zirconium oxides using metal amide precursors. *Chem. Mater.* **14**, 4350–4358 (2002).

11. Lee, S. Y. *et al.* In-situ x-ray photoemission spectroscopy study of atomic layer deposition of TiO2 on silicon substrate. *Jpn. J. Appl. Phys.* **51**, 031102 (2012).

12. Hong, J., Porter, D. W., Sreenivasan, R., McIntyre, P. C. & Bent, S. F. ALD Resist Formed by Vapor-Deposited Self-Assembled Monolayers. *Langmuir* **23**, 1160–1165 (2007).

13. Peng, H., Birkett, G. R. & Nguyen, A. V. The impact of line tension on the contact angle of nanodroplets. *Mol. Simul.* **40**, 934–941 (2014).

14. Kanduč, M., Eixeres, L., Liese, S. & Netz, R. R. Generalized line tension of water nanodroplets. *Phys. Rev. E* **98**, 032804 (2018).

15. Tenney, C. M. & Cygan, R. T. Molecular simulation of carbon dioxide, brine, and clay mineral interactions and determination of contact angles. *Environ. Sci. Technol.* **48**, 2035–2042 (2014).

16. Kanduč, M. Going beyond the standard line tension: Size-dependent contact angles of water nanodroplets. *J. Chem. Phys.* **147**, 174701 (2017).

17. Liu, P., Harder, E. & Berne, B. J. On the calculation of diffusion coefficients in confined fluids and interfaces with an application to the liquid− vapor interface of water. *J. Phys. Chem. B* **108**, 6595–6602 (2004).

18. Elsherbini, A. I. & Jacobi, A. M. Retention forces and contact angles for critical liquid drops on non-horizontal surfaces. *J. Colloid Interface Sci.* **299**, 841–849 (2006).

19. Huhtamäki, T., Tian, X., Korhonen, J. T. & Ras, R. H. A. Surface-wetting characterization using contact-angle measurements. *Nat. Protoc.* **13**, 1521–1538 (2018)

20. Emami, F. S. *et al.* Force Field and a Surface Model Database for Silica to Simulate Interfacial Properties in Atomic Resolution. *Chem. Mater.* **26**, 2647–2658 (2014).

21. Roscioni, O. M., Muccioli, L., Mityashin, A., Cornil, J. & Zannoni, C. Structural Characterization of Alkylsilane and Fluoroalkylsilane Self-Assembled Monolayers on SiO2 by Molecular Dynamics Simulations. *J. Phys. Chem. C* **120**, 14652–14662 (2016).
